# Mutational mechanisms of amplifications revealed by analysis of clustered rearrangements in breast cancers

**DOI:** 10.1093/annonc/mdy404

**Published:** 2018-09-25

**Authors:** D Głodzik, C Purdie, I H Rye, P T Simpson, J Staaf, P N Span, H G Russnes, S Nik-Zainal

**Affiliations:** 1 Division of Oncology and Pathology, Department of Clinical Sciences Lund, Lund University, Lund, Sweden; 2 Wellcome Trust Sanger Institute, Hinxton, Cambridge; 3 Department of Pathology, Ninewells Hospital & Medical School, Dundee, UK; 4 Department of Cancer Genetics, Institute for Cancer Research, Oslo University Hospital, Oslo, Norway; 5 Centre for Clinical Research, Faculty of Medicine, The University of Queensland, Brisbane, Australia; 6 Department of Radiation Oncology, Department of Laboratory Medicine, Radboud University Medical Center, Nijmegen, The Netherlands; 7 Department of Pathology, Oslo University Hospital, Oslo, Norway; 8 Academic Department of Medical Genetics, The Clinical School University of Cambridge, Cambridge, UK

**Keywords:** breast cancer, genome, rearrangements, mutational mechanism, selection, amplification

## Abstract

**Background:**

Complex clusters of rearrangements are a challenge in interpretation of cancer genomes. Some clusters of rearrangements demarcate clear amplifications of driver oncogenes but others are less well understood. A detailed analysis of rearrangements within these complex clusters could reveal new insights into selection and underlying mutational mechanisms.

**Patients and methods:**

Here, we systematically investigate rearrangements that are densely clustered in individual tumours in a cohort of 560 breast cancers. Applying an agnostic approach, we identify 21 hotspots where clustered rearrangements recur across cancers.

**Results:**

Some hotspots coincide with known oncogene loci including *CCND1, ERBB2, ZNF217, chr8:ZNF703/FGFR1, IGF1R*, and *MYC*. Others contain cancer genes not typically associated with breast cancer: *MCL1*, *PTP4A1*, and *MYB*. Intriguingly, we identify clustered rearrangements that physically connect distant hotspots. In particular, we observe simultaneous amplification of *chr8:ZNF703/FGFR1* and *chr11:CCND1* where deep analysis reveals that a chr8–chr11 translocation is likely to be an early, critical, initiating event.

**Conclusions:**

We present an overview of complex rearrangements in breast cancer, highlighting a potential new way for detecting drivers and revealing novel mechanistic insights into the formation of two common amplicons.

**Key Message**

Complex chromosomal rearrangements in breast cancer recur across patients in 21 genomic locations. These ‘hotspots’ contain known oncogenic drivers and putative new driver loci. Detailed analysis of rearrangements at these hotspots highlights chromosomal aberrations likely driven by selection and analysis reveals the underlying mutational processes.

## Background 

Extensive copy number characterisation using comparative genomic hybridisation (CGH) technology has led to remarkable insights into the somatic genetics of breast cancer, including identification of recurrent whole arm gains and losses, homozygous deletions (e.g. *CDKN2A/B*, *PTEN*) and large, common, recurrent driver amplifications (e.g. *ERRB2*, *CCND1*) [1–4]. Despite the increasing resolution provided by CGH technology, there remains a limit to the resolution of detection of copy number aberrations (CNAs) of several hundred kilobases (kb) (supplementary Figure S1, available at *Annals of Oncology* online) [5]. However, CNAs are demarcated by rearrangements that can be detected from whole-genome sequences even when the size of the abnormal copy number segment is as small as 1 kb.

Somatic rearrangements are extremely diverse. Inter-patient variation exists in the quantity, type and distribution of somatic rearrangements even in cancers of the same tissue type [6, 7] and the consequences of rearrangements can also vary considerably. Solitary or low numbers of rearrangement breakpoints may directly confer selective advantage; for example breakpoints that transect tumour suppressor genes or that generate in-frame gene fusion events, such as *ETV6-NTRK3* in breast cancer and *TMPRSS-ERG* fusions in prostate cancer [8, 9]. Collections of breakpoints can reflect driver amplifications. They can also be markers of complex, stochastic chromosomal events (e.g. chromoplexy, chromothripsis) [7, 10, 11] and provide increased resolution in studying mechanisms underpinning CNAs, for example, revealing that breakage-fusion bridge sometimes underpins the formation of the ERBB2 amplicon [12].

## Methods

Recently, 560 whole-genome sequenced breast cancers were expansively curated for somatic mutations, including rearrangements [5]. We previously defined ‘clustered’ rearrangements as clusters of breakpoints that occurred at high density in individual cancer genomes (see supplementary Methods, available at *Annals of Oncology* online). In the current study, we focus on characteristics of clustered rearrangements in 560 breast cancers that so far remained unexplored. In order to assess the impact of clustered rearrangements on breast cancer, we identified chromosomal hotspots where clustered rearrangements recurred in samples from different patients. Using the Piecewise-Constant-Fitting (PCF) algorithm [13] (see supplementary Methods, available at *Annals of Oncology* online), we sought genomic segments where groups of rearrangements exhibited short inter-mutation distances, indicative of ‘hotspots’ that are more frequently rearranged than the background rate. Using this method, we identified highly rearranged genomic loci that recurred in breast cancers. These sites make important contributions to tumorigenesis and reveal mechanisms underpinning chromosomal instability.

## Results

### PCF-based method identifies 21 hotspots of clustered rearrangements across 560 breast cancers

There were 624 clusters of rearrangements in individual breast cancer genomes, comprising 17 247 intra-chromosomal rearrangements, and 6509 inter-chromosomal translocations. Clusters of rearrangements were common: 372 of 560 samples had at least one and were almost as frequent in triple-negative breast cancers (0.96 rearrangement clusters per sample) as in oestrogen receptor (ER)-positive breast cancers (1.00 rearrangement clusters per sample). Among PAM50 subtypes, luminal A cancers had fewest rearrangement clusters per sample (0.6, 95% Poisson CI 0.5–0.9) compared with other subtypes (luminal B 1.2, CI 1.0–1.5 and basal 1.2, CI 1.0–1.5).

**Figure 1.**
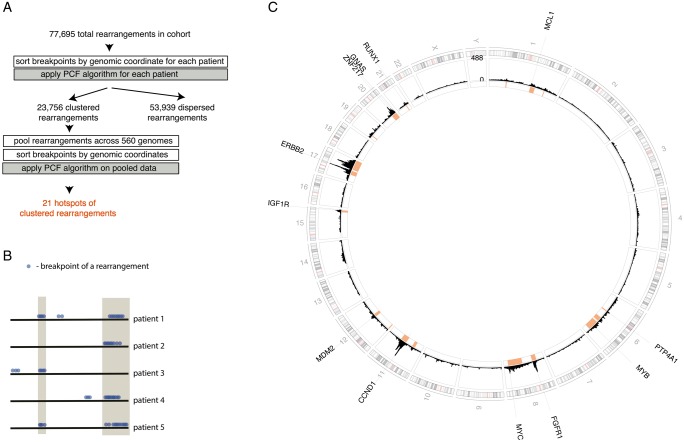
Identification of hotspots of clustered rearrangements in breast cancers. (A) Workflow. (B) Schematic of clusters of rearrangements in individual samples, some of which form hotspots (grey shading). (C) Chromosomal localisation of breakpoints of rearrangements across 560 breast cancer genomes shown as counts (1 Mb bins). Chromosomes are depicted around the outside of the circle. Twenty-one hotspots of clustered rearrangements are shown in red. Positions of genes within each hotspot are indicated. Supplementary Figure S2, available at *Annals of Oncology* online shows a weighted histogram which is robust with respect to extremely rearranged individual samples.

To identify loci where clusters of rearrangements recur *across* multiple independent tumour samples, we pooled all breakpoints in the ‘clustered’ category and sorted them according to position in the reference genome. PCF was applied to find hotspot regions in the genome that are recurrently affected by clusters of breakpoints in multiple patients (Figure 1A and B for workflow).

In all, 21 such hotspots of clustered rearrangements were identified (Figure 1C, supplementary Table S1 and Figures S2 and S3, available at *Annals of Oncology* online), encompassing 8% of the genome, but involving 46% of all breakpoints of clustered rearrangements.

### Recurrent clustered rearrangements identify common, large driver amplicons as well as rare, smaller amplicons

Breakpoint densities for each of the 21 hotspots of clustered rearrangements identified in chromosomes 1, 6, 8, 11, 12, 15, 17, 19, 20 and 21 ranged between 35 and 165 breakpoints per Mb. We expected to find common driver amplification regions such as *CCND1, ERBB2, ZNF217*, *chr8:ZNF703/FGFR1, IGF1R*, and *MYC* as sites of clustered rearrangements recurring across many patients (Figure 1C). These were identified without exception. Hotspots were also identified at *GNAS*, *RUNX1*, and *MDM2*, all recognised as breast cancer genes, even if less frequent.

Interestingly, several hotspots of clustered rearrangements were found near oncogenes that are not typically associated with breast cancer. Curation revealed that a subset had focal copy number gains typical of driver amplicons, albeit on a smaller scale (supplementary Figure S4, available at *Annals of Oncology* online). These hotspots at or near *MCL1* (5.7% samples, 2.7% resulting in *MCL1* amplification), *PTP4A1* (4.5% samples, 1.25% *PTP4A1* amplification) and *MYB* (6.3%, 1.4% *MYB* amplification) occurred at lower frequencies than that of common breast cancer amplicons (supplementary Figures S5 and S6 and Note 1, available at *Annals of Oncology* online for gene expression analysis). Further experiments will be required to verify whether these rarer, smaller and more modest amplicons are indeed driver events.

### Co-occurring hotspots: Inferring co-evolution through detailed breakpoint analyses

Apart from an increased resolution in identifying copy number changes, whole-genome sequencing provides information to base-pair level about direct, physical connections between disparate genomic locations. Each of the 21 hotspots was identified independently through an agnostic approach. If we find that different hotspots are co-occurring at a higher frequency than would be expected, and further are physically connected to each other, this would suggest co-evolution of those allegedly independent hotspots, regardless of their original location on chromosomes. Below we report on two observations—an intra-chromosomal and an inter-chromosomal example—that provide insights into putative drivers and mutational mechanisms.

### Co-evolving clusters on chromosome 6: possible driver loci?

**Figure 2.**
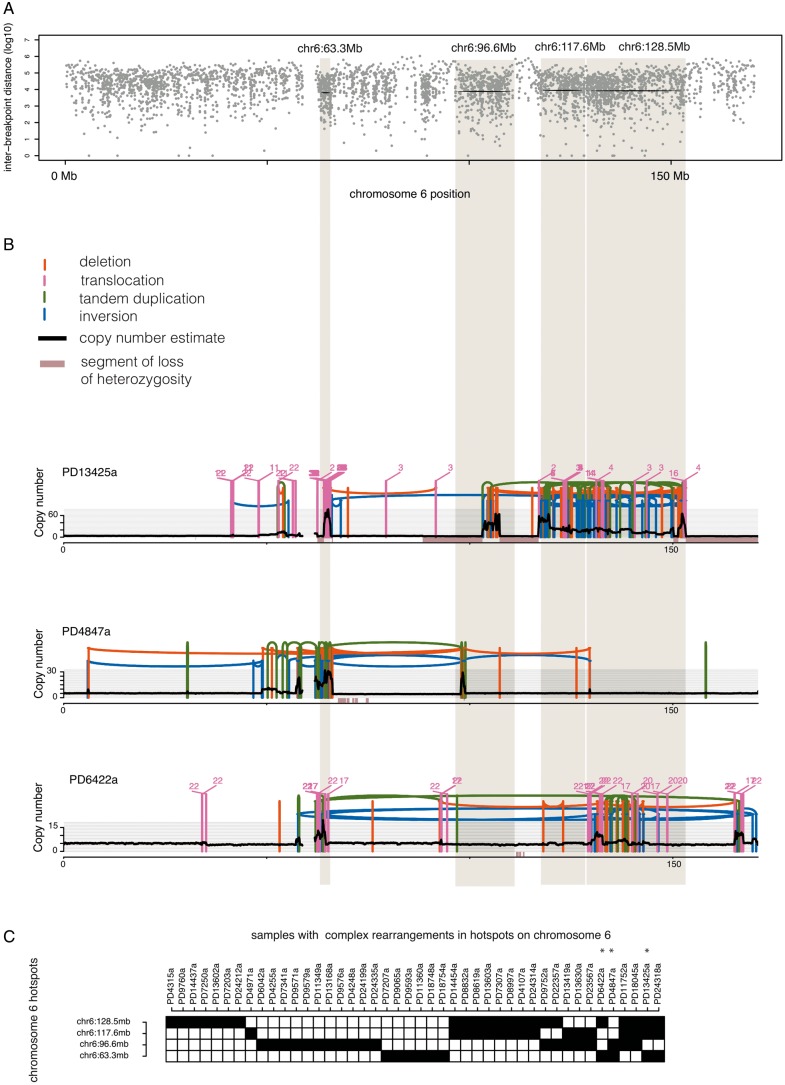
Clustered rearrangements on chromosome 6. (A) Four hotspots identified on chromosome 6 are indicated by the black lines and grey shading. (B) Examples of samples with clustered rearrangements affecting multiple hotspots on chromosome 6: PD13425a, PD4847a, PD6422a. Copy number (*y*-axis) depicted as black dots (10-kb bins, see online methods for details of copy number estimation). Lines represent rearrangements breakpoints (green = tandem duplications, red = deletions, blue = inversions, pink = inter-chromosomal translocations). Pink numbers above breakpoints indicate chromosome where second breakpoint of translocations were found. (C) Matrix indicating samples with clustered rearrangements (in black) focusing on the four hotspots, with samples rearranged in multiple hotspots to the right—samples shown in detail in (B) are highlighted with asterisks.

Four distinct hotspots of clustered rearrangements were identified on chromosome 6; the small amplicon attributed to *PTP4A1* (chr6: 63.3Mb) and three larger hotspots at chr6: 96.6Mb, chr6: 117.6Mb and chr6: 128.5Mb (Figure 2A, supplementary Figure S6, available at *Annals of Oncology* online). Although they are independently identified loci, first we found that the four hotspots occurred together in different combinations in 10 samples (1.7% of cohort, Figure 2C). Second, they were also frequently physically linked through intra-chromosomal rearrangements indicating that they arose or evolved together during tumorigenesis (Figure 2B).

If recurrence of rearrangement clusters is an indicator of putative driver events, then co-occurrence of such hotspots would further contribute to the possibility that they are under selective pressure.

### Co-evolving chr8:ZNF703/*FGFR1* and *CCND1* amplicons reveal two chromosome fusions underpinning amplicon formation

The *chr8:ZNF703/FGFR1* and *chr11:CCND1* amplifications are amongst the most frequent in breast cancer, particularly in ER-positive breast tumours (19% and 28%, respectively, of amplifications in ER-positive tumours; 11% and 16%, respectively, of total cohort). These amplifications have been described before to occur more frequently together in breast cancers, than expected [14]. However, the mechanism underlying these co-occurring amplifications remains uncertain with diverse structural outcomes reported previously [15].

Here, in agreement with previous reports [14], we find co-occurrence of the amplifications of *chr8:ZNF703/FGFR1* and *chr11:CCND1* in 26 patients (5% of total cohort), a frequency higher than expected than if they were independent events (Fisher’s exact test, *P *=* *1.9e^−5^, supplementary Table S2, available at *Annals of Oncology* online).

**Figure 3.**
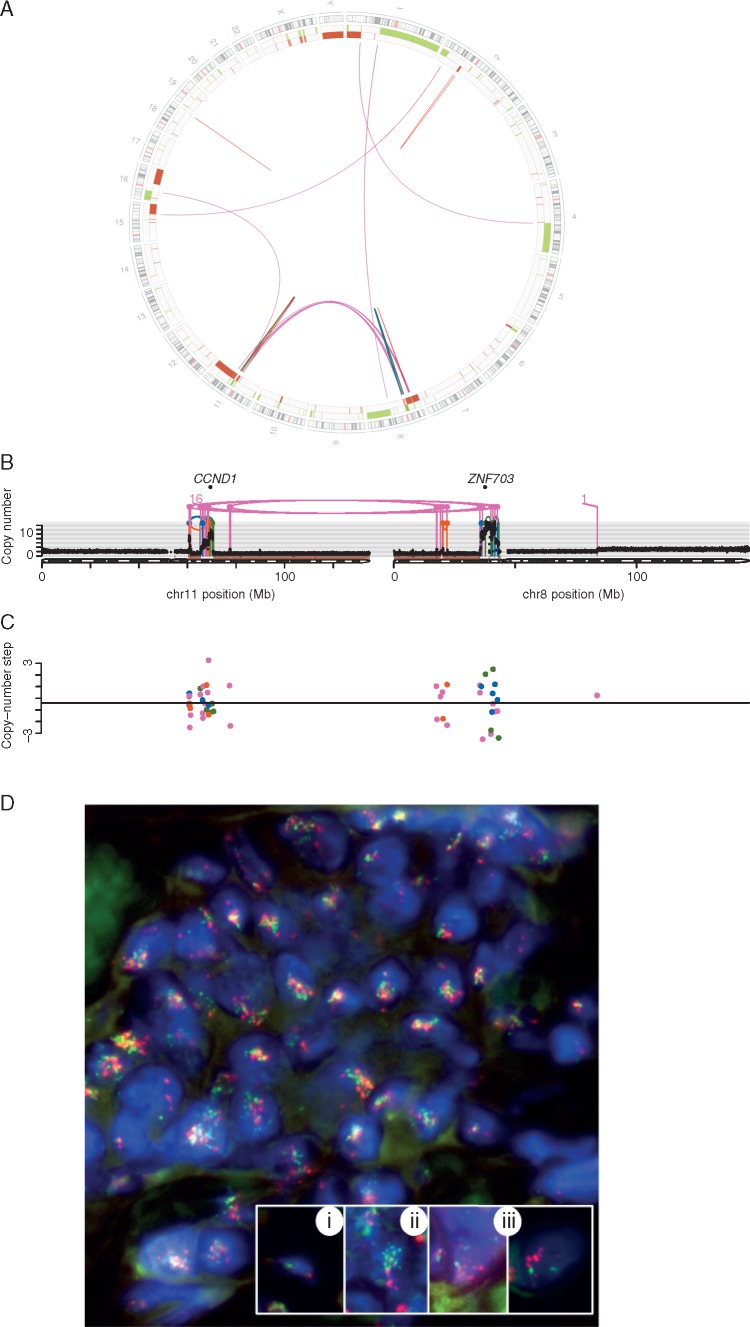
Analysis of *FGFR1* and *CCND1* co-amplification in sample PD4965a. (A) Circos plot showing rearrangements, loss of heterozygosity (LOH) (red) and copy number gain (green). Chromosomal ideogram depicted on outermost circle. Lines in the centre of the circle show rearrangements (colours described in Figure 2). (B) Representation of two chromosomes connected by translocations. Copy number (*y*-axis) and lines represent rearrangements breakpoints described as in Figure 2. (C) Copy number changes at rearrangement breakpoints (dot = rearrangements breakpoint, colours as described in Figure 2), with biggest differences at translocation breakpoints. (D) FISH analysis of the same tumour (bottom): frozen sections of tumour were probed for *CCND1* (red) and *FGFR1* (green) and the respective chromosome centromeres. Subset (i) shows a normal cell nucleus with a single copy of both genes. Subset (ii) shows a tumour cell nucleus with multiple *FGFR1* (green) signals (average 14.9/nucleus when counting 60 nuclei) and two chromosome 8 centromere (red) signals. Subset (iii) shows two tumour cell nuclei with multiple *CCND1* (red) signals (average 10.4/nucleus when counting 60 nuclei) and two chromosome 11 centromere (green) signals. Main FISH image shows combination of CCND1 (red) and FGFR1 (green) probes across multiple tumour cell nuclei. Note significant co-amplification of both genes and clustering of signals indicating co-evolving of the amplification/translocation in this case.

**Figure 4.**
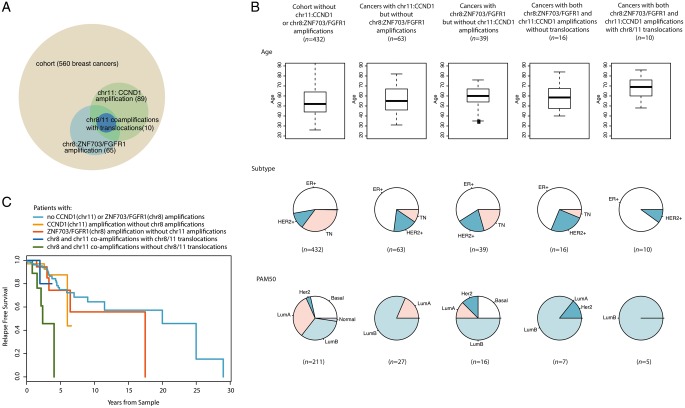
Comparison of clinical data for groups of patients with *chr8:ZNF703/FGFR1* and *chr11:CCND1* amplifications. (A) Venn diagram for groups of patients defined by amplifications and chr8/11 translocations. (B) Age, breast cancer subtype and PAM50 classification of the patient groups. (C) Relapse-free survival for the patient groups. Different age distributions and therapies between groups confound this comparison.

Furthermore, we detect translocation breakpoints connecting the *chr8:ZNF703/FGFR1* and *chr11:CCND1* amplicons in 11 out of 26 patients with co-occurring amplifications (42%; Figure 3 for a detailed analysis of a single sample), showing that these amplicons are often physically connected. This phenomenon of co-localising *chr8:ZNF703/FGFR1* and *chr11:CCND1* amplicons tends to be seen in ER-positive tumours, among patients diagnosed at an older age (Figure 4).

To confirm physical proximity of the *chr8:ZNF703/FGFR1* and *chr11:CCND1* amplicons, FISH analysis was carried out on four samples. Nuclear co-localisation of the *chr8:ZNF703/FGFR1* and *chr11:CCND1* amplicons was observed. In three samples, the counts of co-amplified signals were sufficient to confidently establish co-localisation of the amplicons in nuclei (Figure 3D, supplementary Figure S7, available at *Annals of Oncology* online). The co-localisation of the amplified sequences confirms linked co-evolution of amplicons that were originally located on separate chromosomes.

**Figure 5.**
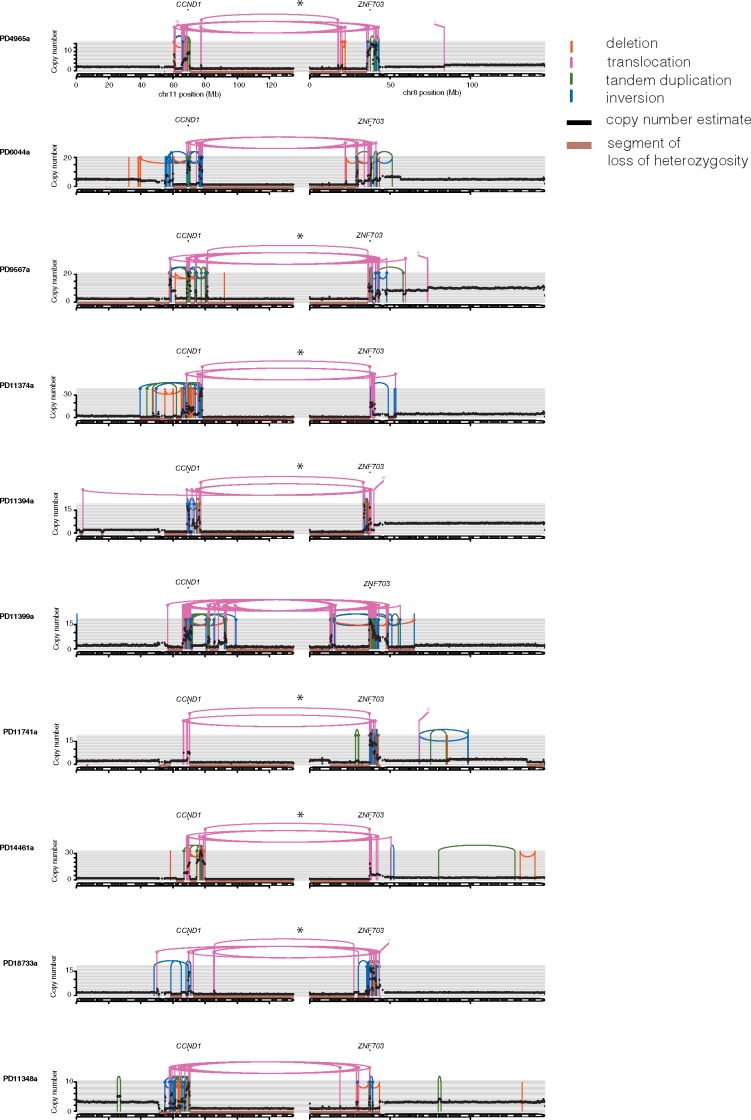
Representation of ten samples with *FGFR1/CCND1* amplifications and translocations between the two amplicons. Copy number (*y*-axis) and lines represent rearrangements as described in Figure 2. Translocations joining the lowest coordinates of *chr8:ZNF703/FGFR1* amplicon to the highest coordinate of *chr11:CCND1* amplicon highlighted with asterisks. Segments of LOH are marked in pale red colour.

In 10 samples, the translocations only connect chromosomes 8 and 11 (Figure 5), while in the remaining sample PD13608a, five other chromosomes were also involved (supplementary Figure S8, available at *Annals of Oncology* online). In 7 out of the 10 samples where only chromosomes 8 and 11 are involved, there is a translocation which joins the lower-most coordinate of the *chr8:ZNF703/FGFR1* amplicon, located on 8p, to the higher-most coordinate of the *chr11:CCND1* amplicon, located on 11q (marked with asterisks in Figure 5). The translocations are associated with chromosomal copy number loss terminal to the breakpoints (seen as 8p and 11q loss-of-heterozygosity respectively marked in pale red in Figure 5). This observation implies that a chr8–chr11 translocation with associated loss of portions of the chromosome terminal to the breakpoint is likely to be an early, critical, initiating event in the tumours where they were found.

Moreover, in the 10 samples, there are many additional chr8/chr11 translocations in each patient, and these additional translocations are distant from the predicted driver amplifications (*ZNF703/FGFR1* and *CCND1*, respectively). Additionally, some intervening sequences were lost. The translocations demarcate borders of chromosomal segments where the difference in total copy number is particularly marked: Figure 3C depicts ‘copy number steps’ at rearrangement breakpoints which are calculated as the absolute difference in total copy number between 5-kb regions to the left and to the right of a breakpoint in the reference genome. In 7 out of the 10 samples, we found that the largest copy number steps were observed at breakpoints of these additional chr8/chr11 inter-chromosomal translocations as compared with intra-chromosomal rearrangements (supplementary Table S3, available at *Annals of Oncology* online). In all 10 samples, there are examples of inter-chromosomal breakpoints with copy number steps of 3 or more. Amplifications of translocation breakpoints are therefore frequent.

To confirm this analytical observation, FISH analysis was carried out on ancillary translocation breakpoints that are distant from the target driver gene in sample PD18733a. The FISH analysis confirmed high amplification of translocation breakpoints and their co-localisation in the nuclei (supplementary Methods and Figure S9, available at *Annals of Oncology* online). In terms of the chronology of events, the amplification of these loci must have occurred after the formation of the multiple translocations between the pairs of same two chromosomes.

**Figure 6.**
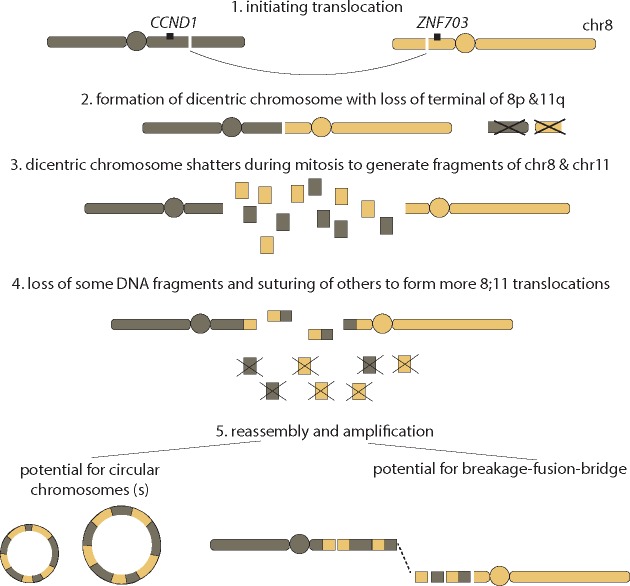
Proposed mechanism for *FGFR1/CCND1* co-amplifications.

We propose the following model: the formation of the *chr8:ZNF703/FGFR1* and *chr11:CCND1* amplicons is initiated by a translocation between 8p and 11q resulting in copy number losses terminal to the translocation breakpoints (Figure 6). A dicentric chromosome is formed that likely shatters during cell division, creating multiple opportunities for further translocation rearrangements to form between pieces of chr8 and chr11. Some intervening genomic pieces may be lost, while some retained and then amplified, producing the patterns of high-level amplification delimited by translocations that we observe to be interspersed by copy number loss/neutral regions in these breast cancers.

### Chromosome arm loss and amplicon formation

According to the model, losses of chromosome arms 8p and 11q precede formation of the amplicons. To further evaluate this hypothesis, we assessed the frequency of 8p and 11q losses across the cohort. Eleven out of thirteen samples with independent amplifications of both, but without rearrangements between the two chromosomes also display loss of 8p and 11q (supplementary Figures S10 and S11, available at *Annals of Oncology* online). Arm losses are often associated with amplifications: out of 274 samples with 8p loss, 168 (61.3%) displayed amplicons elsewhere on the chromosome. Out of 237 samples with 11q loss, 97 (40.9%) displayed *chr11:CCND1* gain. The majority of amplifications are accompanied by adjacent chromosome arm losses, but not all chromosomes with arms losses developed amplifications.

### Is the mutational process unique to co-amplifications of *chr8:ZNF703/FGFR1* and *chr11:CCND1*?

The mechanistic steps underpinning the co-amplifications between chromosomes 8 and 11 could be specific to the two sites or may be a more generalised phenomenon.

To explore this, we searched for other examples of co-occurring driver amplifications and assessed the patterns of rearrangements demarcating them. Co-amplifications of other driver loci in breast cancer are observed, for example *chr20:ZNF217* and *chr8:MYC* is seen in 18 samples (*P *=* *1e^−04^) and *chr20:ZNF217* and *chr8:ZNF703* in 11 samples (*P *=* *9e^−03^) (supplementary Table S1, available at *Annals of Oncology* online). However, these co-amplifications did not show enrichment of translocations between affected chromosomes, nor did they exhibit simultaneous telomeric loss beyond the translocation breakpoint. Thus, although other co-occurring amplifications exist, they do not arise via the same mechanism of fusion of two chromosomes that results in the *chr8:ZNF703/FGFR1* and *chr11:CCND1* co-amplifications.

Finally, we conducted an exhaustive search for clustered rearrangements with similar genomic properties to the chr8–chr11 co-amplifications. We identified six other samples with clustered rearrangements involving two chromosomes that had translocations connecting the two chromosomes, and had adjacent losses of chromosome arms (supplementary Figure S12 and S13, available at *Annals of Oncology* online). Although chromosome 8 or 11 was involved in five of the six identified events, no other chromosomal pair was recurrent unlike in the chr8–chr11 co-amplifications.

## Discussion

### Alternative way of detecting drivers—recurrence and co-occurrence

Traditionally, recurrence of exonic mutations has been used as evidence for selection, particularly for single-base substitutions and frameshifting insertions/deletions. The principle of recurrence has also been used to detect selection for simple somatic rearrangements [16]. Here, we describe recurrent clustered rearrangements of a more complex nature; some of which span multiple regions on single or multiple chromosomes. Detailed analyses of the rearrangements forming these complex events suggest a role of selection in their formation. We observed co-evolution of clustered rearrangements recurrently affecting disparate hotspot regions on a single chromosome (e.g. chromosome 6), as well as on different chromosomes (e.g. co-amplifications of chromosomes 8 and 11). We posit that such chromosomal events are unlikely to occur by chance.

### Rare amplicons detected by analysis of clustered rearrangements

In addition to known driver amplicons, we identified novel regions of the genome that are recurrently affected by clustered rearrangements, albeit at moderate frequency. Some of these events increase the number of copies of oncogenes, but further functional work is required to demonstrate whether they are driver events in breast cancer.

### Deep analysis of co-occurrence reveals novel mutational mechanisms

The processes that lead to co-occurring amplifications of the *chr8:ZNF703/FGFR1* and *chr11:CCND1* loci are particularly intriguing, as we identified recurrent translocations between the two regions suggesting co-evolution. Indeed, the fusion of two chromosomes appears to be the initiating mechanistic event in a substantial proportion of these tumours. The co-evolution of the *chr8:ZNF703/FGFR1* and *chr11:CCND1* amplicons has been subject of controversy in the literature [14, 15]. Among tumours with co-amplifications in our cohort, there are examples of independent evolution and of linked co-evolution. The novel mutational process that is described here directly contributes towards the high frequency of the co-amplifications observed in breast cancers.

### Conclusions

Clustered rearrangements are common in breast cancer genomes, and often associated with gene amplifications that drive oncogenesis. Understanding the process of amplicon formation, an example of which we present here for the *chr8:ZNF703/FGFR1* and *chr11:CCND1* co-amplifications, will be important for our understanding of the origins of a subset of breast cancers. 

## Supplementary Material

Supplementary Data
