# Supplementary material for: Mutational mechanisms of amplifications revealed by analysis of clustered rearrangements in breast cancers
Source: Ann Oncol. 2018 Sep 25;29(11):2223–31. doi: 10.1093/annonc/mdy404 (PMC6290883; doi:10.1093/annonc/mdy404)
Supplement: Supplementary Data [file mdy404_supp.zip › mdy404-suppl_data/mdy404_All_Main_Figures.pdf]

Figure 1

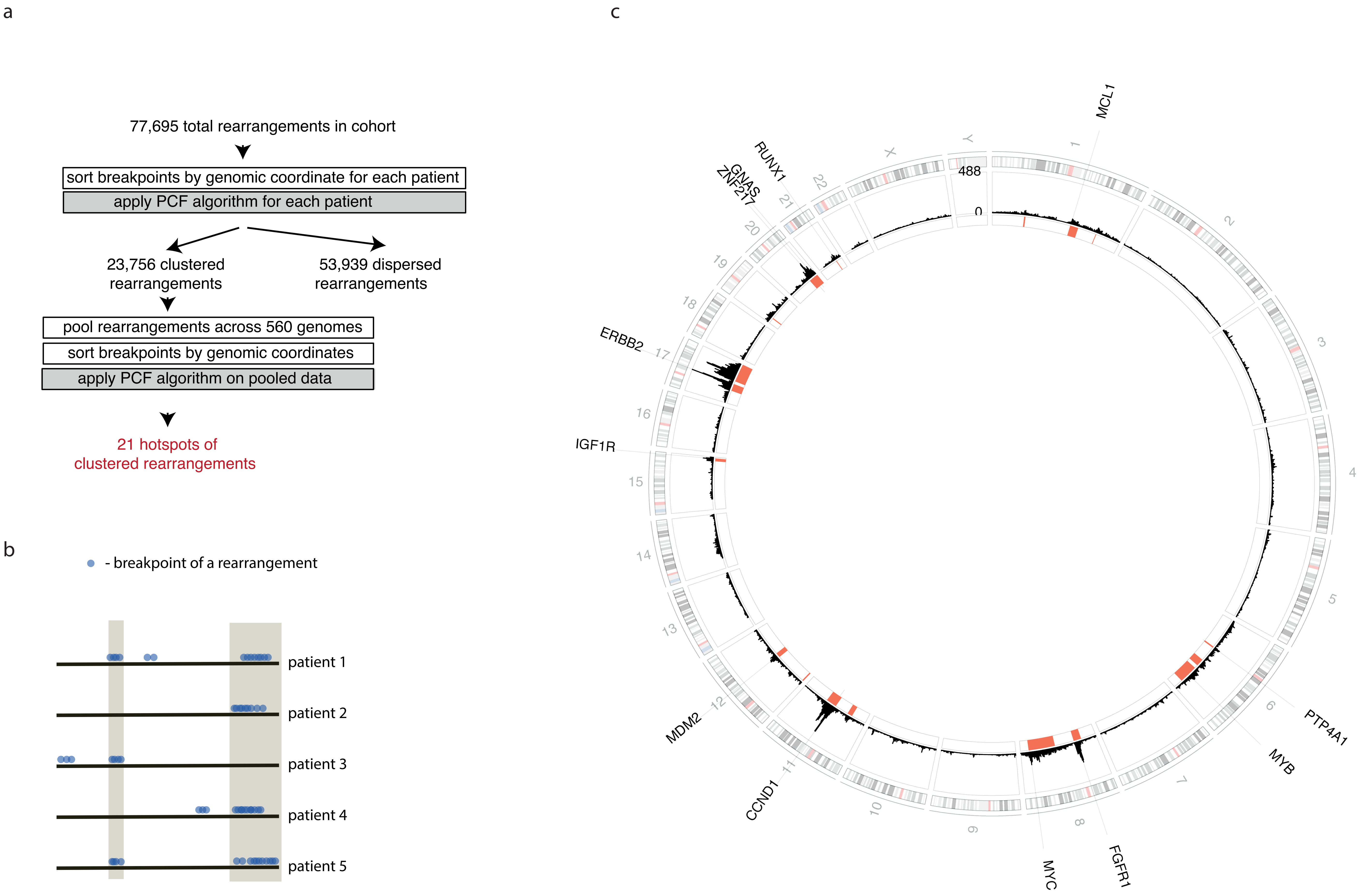

a
77,695 total rearrangements in cohort
sort breakpoints by genomic coordinate for each patient
apply PCF algorithm for each patient
23,756 clustered rearrangements
53,939 dispersed rearrangements
pool rearrangements across 560 genomes
sort breakpoints by genomic coordinates
apply PCF algorithm on pooled data
21 hotspots of clustered rearrangements
b
- breakpoint of a rearrangement
patient 1
patient 2
patient 3
patient 4
patient 5
c
488
0
MCL1
RUNX1
GNAS
ZNF217
ERBB2
IGF1R
MDM2
CCND1
MYC
FGFR1
MYB
PTP4A1
1
2
3
4
5
6
7
8
9
10
11
12
13
14
15
16
17
18
19
20
21
22
X
Y

Figure 2

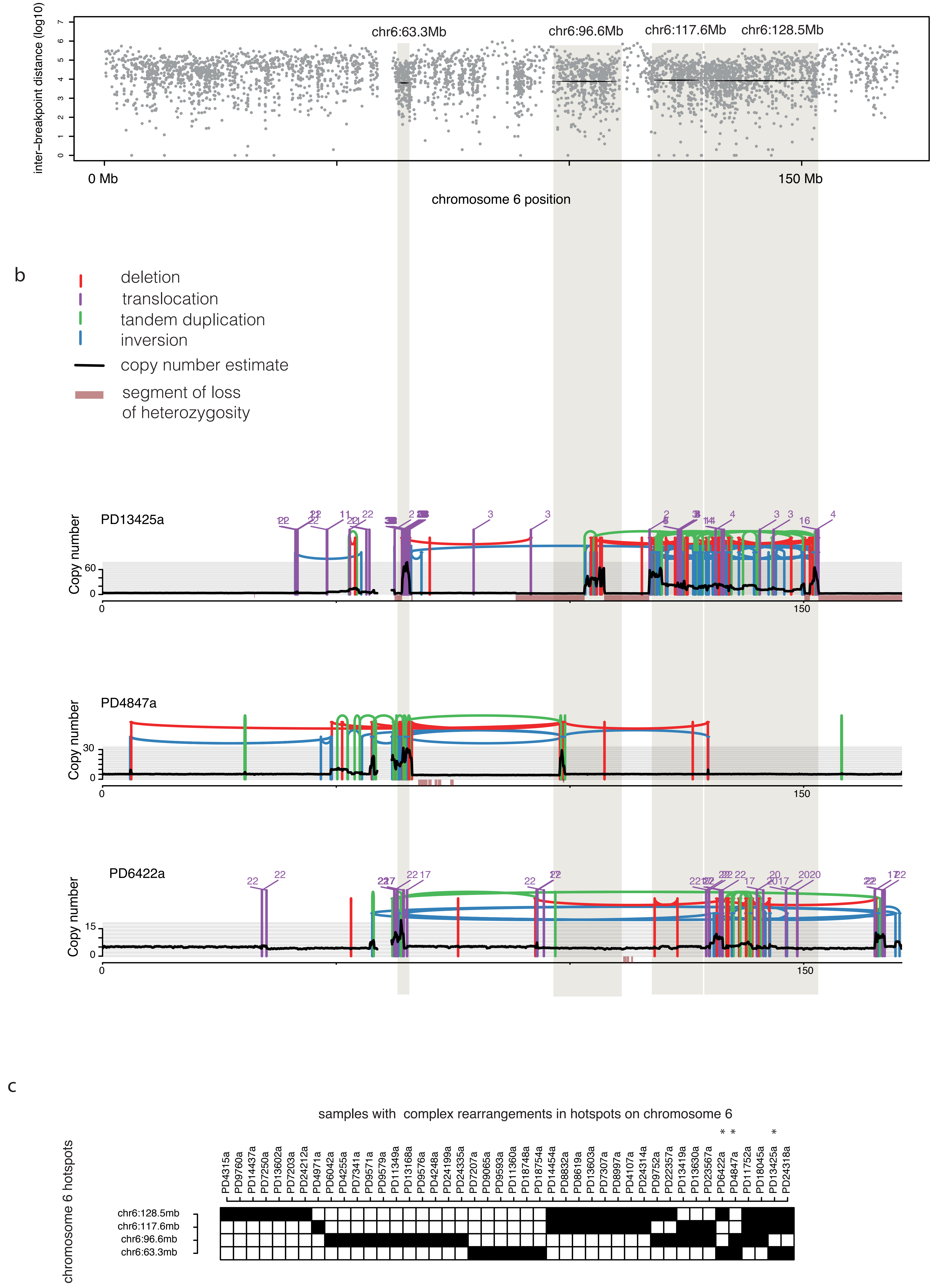
a
inter−breakpoint distance (log10)
chr6:63.3Mb
chr6:96.6Mb
chr6:117.6Mb
chr6:128.5Mb
0 Mb
150 Mb
chromosome 6 position
b
deletion
translocation
tandem duplication
inversion
copy number estimate
segment of loss of heterozygosity
PD13425a
PD4847a
PD6422a
Copy number
c
samples with complex rearrangements in hotspots on chromosome 6
PD4315a PD9760a PD14437a PD7250a PD13602a PD7203a PD24212a PD4971a PD6042a PD4255a PD7341a PD9571a PD9579a PD11349a PD13168a PD9576a PD42448a PD24199a PD24335a PD7207a PD9065a PD9593a PD11360a PD18748a PD18754a PD14454a PD8832a PD8619a PD13603a PD7307a PD8997a PD4107a PD24314a PD9752a PD22357a PD13419a PD13630a PD23567a PD6422a PD4847a PD11752a PD18045a PD13425a PD24318a
chromosome 6 hotspots
chr6:128.5mb
chr6:117.6mb
chr6:96.6mb
chr6:63.3mb

Figure 3

a

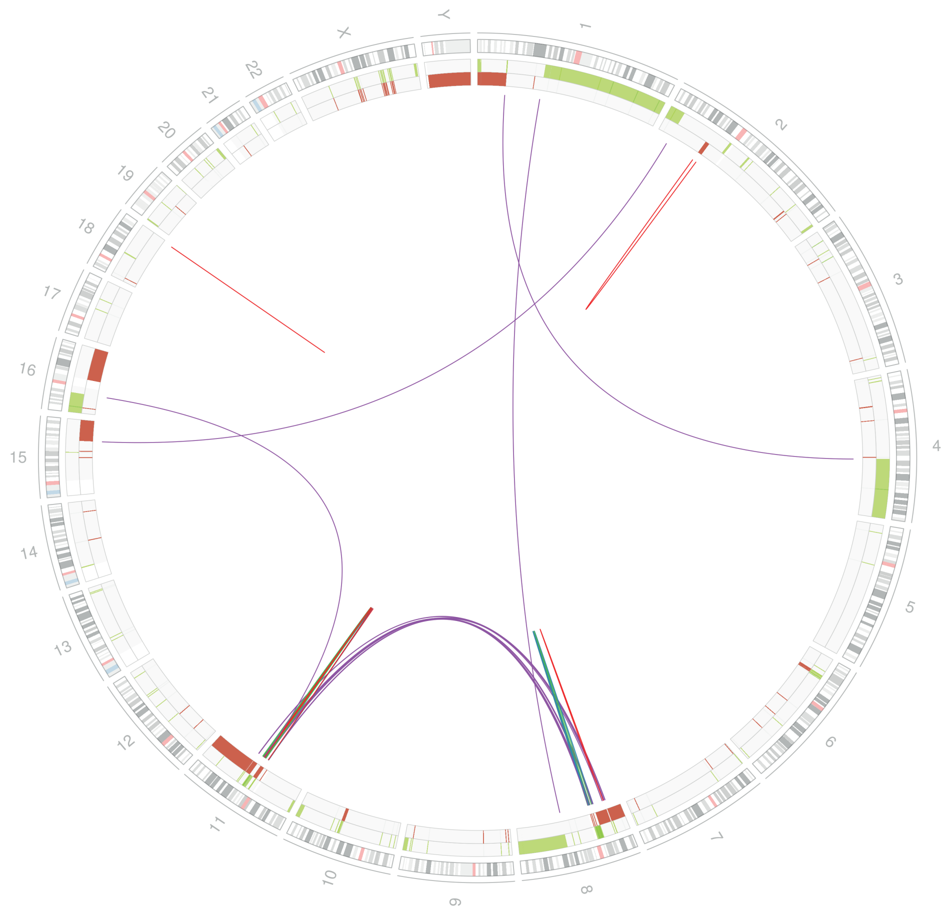

b

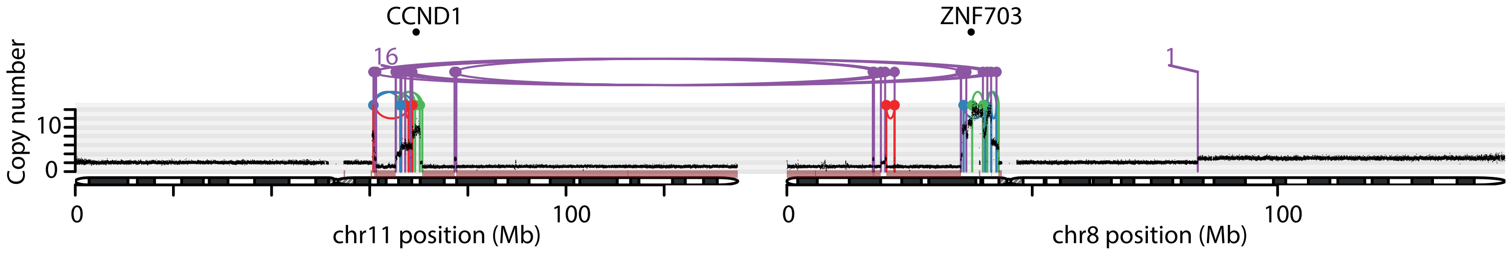

c

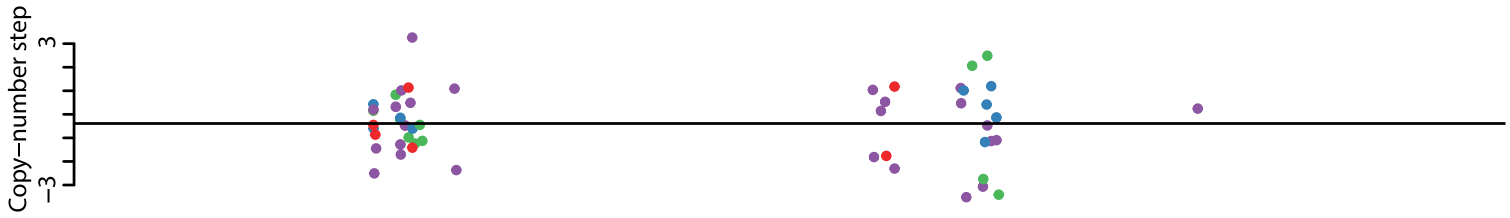

d

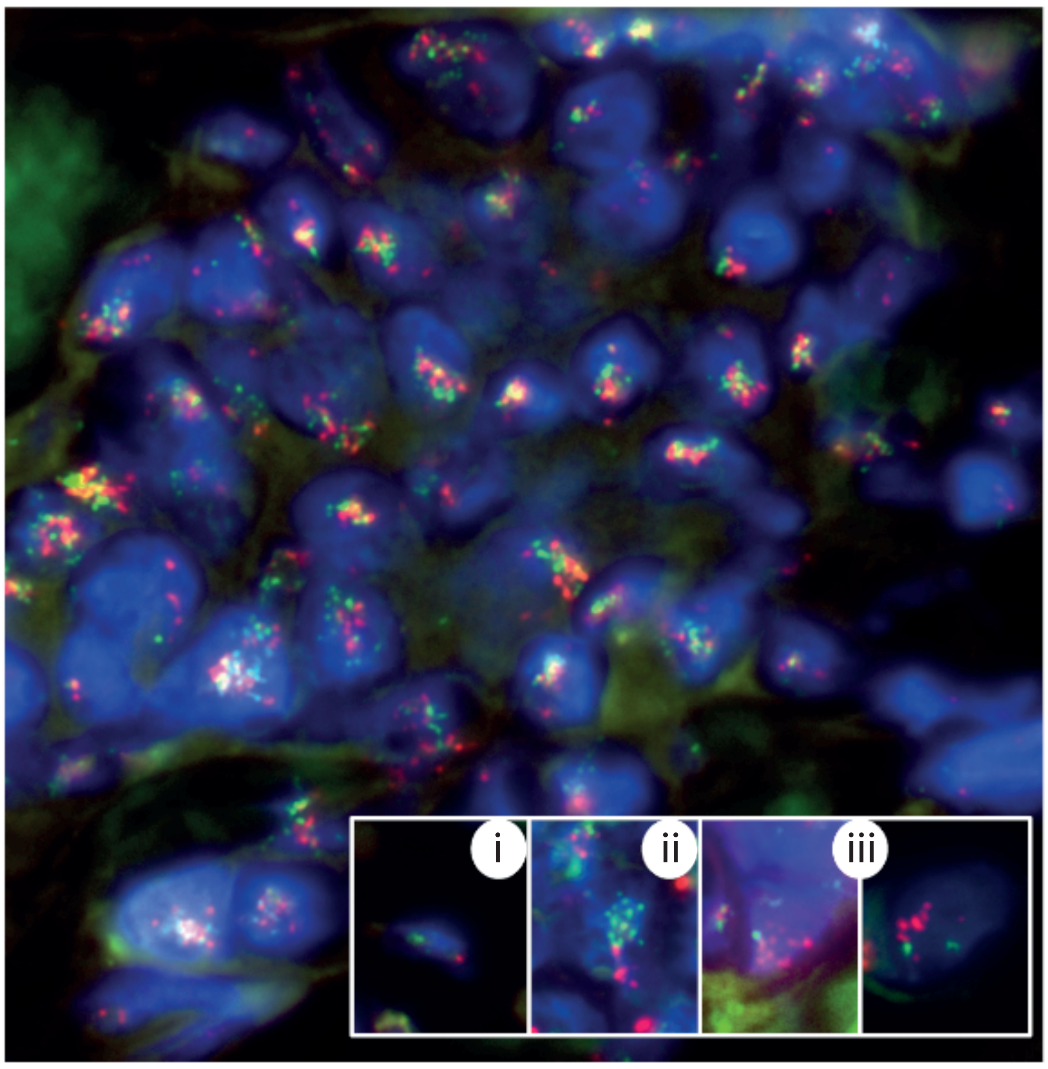

Figure X

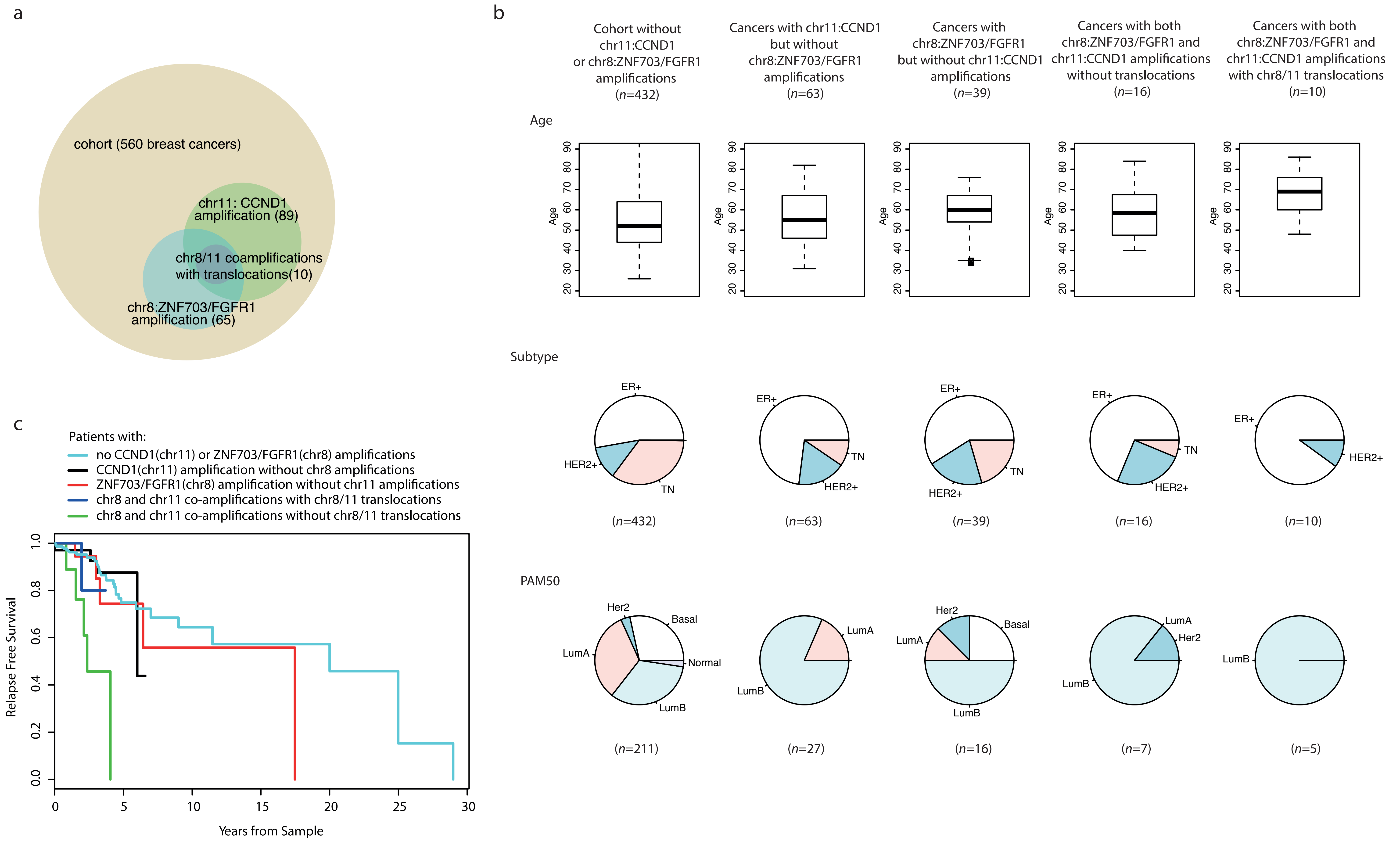

a
cohort (560 breast cancers)
chr11: CCND1 amplification (89)
chr8/11 coamplifications with translocations(10)
chr8:ZNF703/FGFR1 amplification (65)
b
Cohort without chr11:CCND1 or chr8:ZNF703/FGFR1 amplifications (n=432)
Cancers with chr11:CCND1 but without chr8:ZNF703/FGFR1 amplifications (n=63)
Cancers with chr8:ZNF703/FGFR1 but without chr11:CCND1 amplifications (n=39)
Cancers with both chr8:ZNF703/FGFR1 and chr11:CCND1 amplifications without translocations (n=16)
Cancers with both chr8:ZNF703/FGFR1 and chr11:CCND1 amplifications with chr8/11 translocations (n=10)
Age
Subtype
ER+
HER2+
TN
(n=432)
(n=63)
(n=39)
(n=16)
(n=10)
PAM50
Her2
Basal
LumA
LumB
Normal
(n=211)
(n=27)
(n=16)
(n=7)
(n=5)
c
Patients with:
no CCND1(chr11) or ZNF703/FGFR1(chr8) amplifications
CCND1(chr11) amplification without chr8 amplifications
ZNF703/FGFR1(chr8) amplification without chr11 amplifications
chr8 and chr11 co-amplifications with chr8/11 translocations
chr8 and chr11 co-amplifications without chr8/11 translocations
Relapse Free Survival
Years from Sample

Figure 6

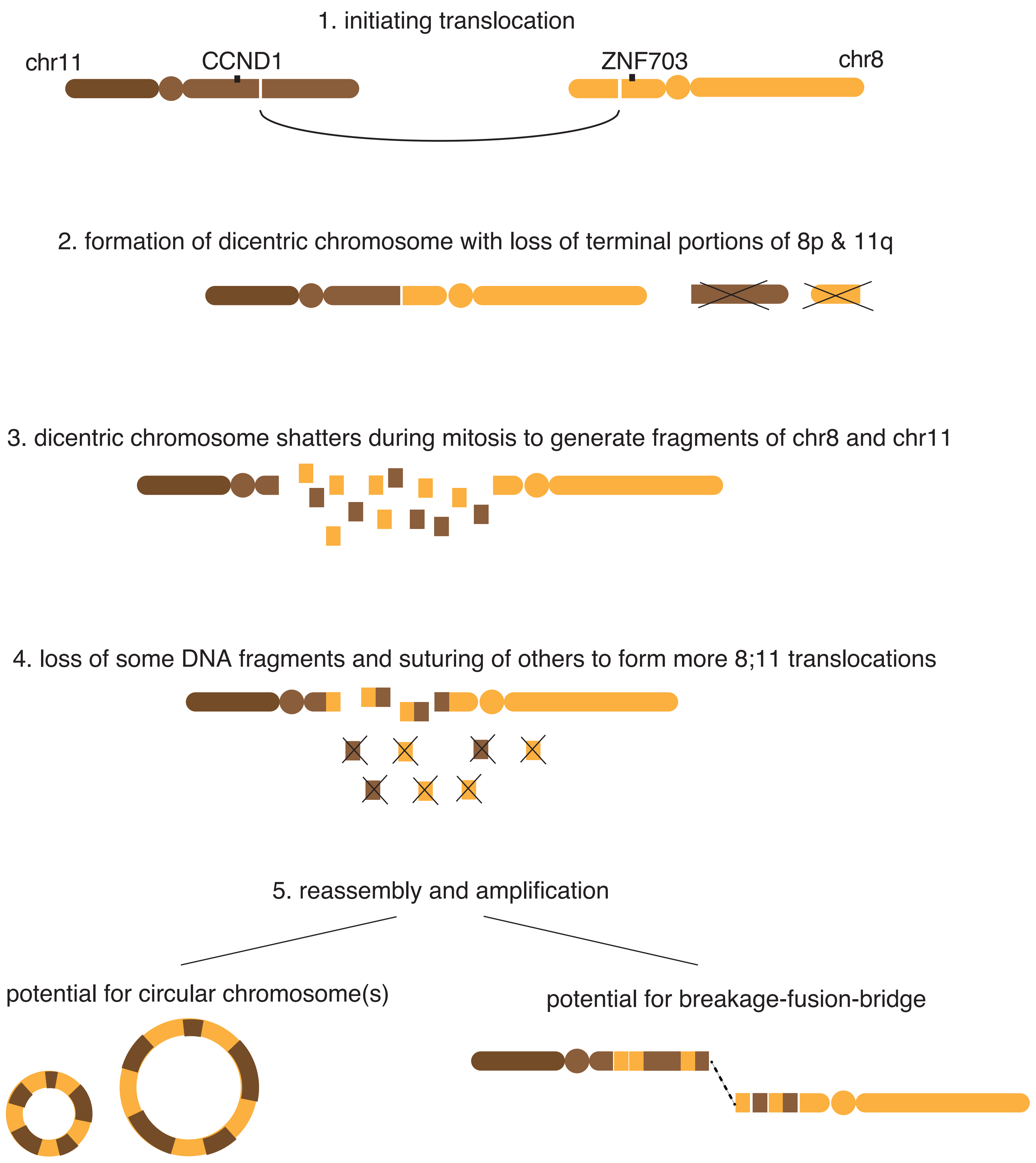

1. initiating translocation
chr11
CCND1
ZNF703
chr8
2. formation of dicentric chromosome with loss of terminal portions of 8p & 11q
3. dicentric chromosome shatters during mitosis to generate fragments of chr8 and chr11
4. loss of some DNA fragments and suturing of others to form more 8;11 translocations
5. reassembly and amplification
potential for circular chromosome(s)
potential for breakage-fusion-bridge
