# Supplementary material for: Mutational mechanisms of amplifications revealed by analysis of clustered rearrangements in breast cancers
Source: Ann Oncol. 2018 Sep 25;29(11):2223–31. doi: 10.1093/annonc/mdy404 (PMC6290883; doi:10.1093/annonc/mdy404)
Supplement: Supplementary Data [file mdy404_supp.zip › mdy404-suppl_data/mdy404_Supplementary_Table_3.docx]

| **Supplementary Table 3** | |  |  |  |  |  |  |  |
| --- | --- | --- | --- | --- | --- | --- | --- | --- |
| **sample** |  |  | Identifier of sample | |  |  |  |  |
| **subtype** |  |  | Cancer subtype from clinical data | | |  |  |  |
| **AIMS subtype** | |  | Cancer subtype inferred from gene expression data | | | |  |  |
| **is.8p.lost** |  |  | whether chromosome 8p is lost, loss-of-heterozygosity | | | | |  |
| **is.11q.lost** |  |  | whether chromosome 11q is lost, loss-of-heterozygosity | | | | |  |
| **max.cn.step.pf** | |  | type of rearrangement with highest copy number step (1 - inverstion, 2 - deletion, 4 - tandem duplication, 32 translocation) | | | | | |
| **max.cn.orientation** | |  | genomic strands from reads reporting the rearrangement with highest copy increase to copy numner between regions on either side of the breakpoint in reference genome | | | | | |
| **is.matching.losses** | |  | whether the lowest-coordinate breakpoint on chromosome 8 is connected to highest coordinate breakpoint on chromosome 11 through translocation | | | | | |
| **orientation.matching** | |  | whether orientation of the rearrangement is consisent | | | | |  |
|  |  |  |  |  |  |  |  |  |
| **sample** | **subtype** | **AIMS subtype** | **is.8p.lost** | **is.11q.lost** | **max.cn.step.pf** | **max.cn.orientation** | **is.matching.losses** | **orientation.matching** |
| PD4965a | ER+ | NA | TRUE | TRUE | 32 | + + | TRUE | - - |
| PD6044a | ER+ | NA | TRUE | TRUE | 32 | + + | FALSE | NA |
| PD9567a | ER+ | LumB | TRUE | TRUE | 2 | NA | TRUE | - - |
| PD11374a | ER+ | LumB | TRUE | TRUE | 32 | - - | TRUE | - - |
| PD11394a | ER+ | LumB | TRUE | TRUE | 32 | + + | TRUE | - - |
| PD11399a | ER+ | LumB | TRUE | TRUE | 32 | - + | FALSE | NA |
| PD11741a | ER+ | NA | TRUE | TRUE | 4 | NA | TRUE | - - |
| PD14461a | ER+ | LumB | TRUE | TRUE | 32 | - - | TRUE | - - |
| PD18733a | ER+ | NA | TRUE | TRUE | 32 | - - | TRUE | - - |
| PD11348a | HER2+ | NA | TRUE | TRUE | 1 | NA | FALSE | NA |
