# Supplementary material for: Mutational mechanisms of amplifications revealed by analysis of clustered rearrangements in breast cancers
Source: Ann Oncol. 2018 Sep 25;29(11):2223–31. doi: 10.1093/annonc/mdy404 (PMC6290883; doi:10.1093/annonc/mdy404)
Supplement: Supplementary Data [file mdy404_supp.zip › mdy404-suppl_data/mdy404_Supplementary_Table_4.docx]

| **Supplementary Table 4** |  |  |  |  |  |  |  |  |  |  |  |  |  |  |  |  |  |
| --- | --- | --- | --- | --- | --- | --- | --- | --- | --- | --- | --- | --- | --- | --- | --- | --- | --- |
| **hotspot** |  | hotspot ID |  |  |  |  |  |  |  |  |  |  |  |  |  |  |  |
| **no.samples** |  | number of samples with rearrangements in the hotspot | | | |  |  |  |  |  |  |  |  |  |  |  |  |
| **assoc.(ploss, pgain, qloss, qgain)** |  | p-values: asociation between rearrangement in hotspot and chromosome arm anomalies | | | |  |  |  |  |  |  |  |  |  |  |  |  |
| **no.samples.(ploss, pgain, qloss, qgain)** |  | number of samples with chormosome anomalies with rearrangements in the hotspot | | |  |  |  |  |  |  |  |  |  |  |  |  |  |
| **no.samples.ploss, pgain, qloss, qgain).out** |  | number of samples with chromosome anomalies without rearrangements in the hotspot | | |  |  |  |  |  |  |  |  |  |  |  |  |  |
| **ploss, pgain, qloss, qgain** |  | enrichment of chromosome arm anomalies in samples with rearrangements | | |  |  |  |  |  |  |  |  |  |  |  |  |  |
|  |  |  |  |  |  |  |  |  |  |  |  |  |  |  |  |  |  |
| **hotspot** | **no.samples** | **assoc.ploss** | **assoc.pgain** | **assoc.qloss** | **assoc.qgain** | **no.samples.ploss** | **no.samples.pgain** | **no.samples.qloss** | **no.samples.qgain** | **no.samples.ploss.out** | **no.samples.pgain.out** | **no.samples.qloss.out** | **no.samples.qgain.out** | **ploss** | **pgain** | **qloss** | **qgain** |
| peak_clust_chr1_60.8mb | 9 | 1.0E+00 | 1.0E+00 | 1.0E+00 | 1.0E+00 | 1 | 1 | 0 | 6 | 99 | 79 | 22 | 250 | 0.6 | 0.7 | 0.0 | 2.4 |
| peak_clust_chr1_150mb | 32 | 1.0E+00 | 1.0E+00 | 1.0E+00 | 1.0E+00 | 4 | 5 | 2 | 26 | 68 | 78 | 25 | 332 | 1.0 | 1.1 | 1.3 | 2.6 |
| peak_clust_chr1_201.3mb | 9 | 1.0E+00 | 1.0E+00 | 5.0E-01 | 1.0E+00 | 2 | 3 | 3 | 4 | 52 | 97 | 31 | 363 | 2.7 | 2.3 | 8.4 | 0.4 |
| peak_clust_chr6_63.3mb | 25 | 1.0E+00 | 1.0E+00 | 6.9E-05 | 1.0E+00 | 3 | 9 | 18 | 7 | 56 | 113 | 134 | 99 | 1.2 | 2.1 | 7.7 | 1.7 |
| peak_clust_chr6_96.6mb | 33 | 1.0E+00 | 1.0E+00 | 1.0E+00 | 1.0E+00 | 8 | 4 | 11 | 9 | 86 | 107 | 153 | 99 | 1.7 | 0.5 | 1.2 | 1.6 |
| peak_clust_chr6_117.6mb | 28 | 1.0E+00 | 1.0E+00 | 1.0E+00 | 1.0E+00 | 7 | 3 | 11 | 7 | 107 | 101 | 157 | 106 | 1.4 | 0.5 | 1.5 | 1.3 |
| peak_clust_chr6_128.5mb | 35 | 1.0E+00 | 1.0E+00 | 9.8E-01 | 1.0E+00 | 11 | 7 | 19 | 8 | 104 | 97 | 168 | 97 | 2.0 | 1.1 | 2.5 | 1.3 |
| peak_clust_chr8_28.6mb | 76 | 1.3E-09 | 1.0E+00 | 1.0E+00 | 7.1E-06 | 65 | 9 | 0 | 63 | 209 | 86 | 21 | 238 | 9.7 | 0.6 | 0.0 | 5.0 |
| peak_clust_chr8_80.2mb | 80 | 5.3E-05 | 1.0E+00 | 4.2E-02 | 1.0E+00 | 23 | 28 | 12 | 48 | 42 | 189 | 21 | 277 | 4.9 | 0.8 | 3.9 | 1.1 |
| peak_clust_chr11_28.7mb | 25 | 1.0E+00 | 1.0E+00 | 1.0E+00 | 1.0E+00 | 9 | 5 | 13 | 4 | 87 | 90 | 149 | 75 | 3.0 | 1.2 | 2.8 | 1.2 |
| peak_clust_chr11_65.1mb | 114 | 1.0E+00 | 1.0E+00 | 4.5E-21 | 1.0E+00 | 14 | 19 | 95 | 9 | 61 | 72 | 141 | 74 | 1.1 | 1.0 | 10.8 | 0.4 |
| peak_clust_chr12_0.1mb | 10 | NA | 1.0E+00 | 1.0E+00 | 1.0E+00 | NA | NA | 2 | 4 | NA | NA | 94 | 107 | #VALUE! | #VALUE! | 1.2 | 2.8 |
| peak_clust_chr12_65.1mb | 23 | 1.0E+00 | 1.0E+00 | 2.7E-01 | 7.7E-02 | 5 | 8 | 11 | 12 | 65 | 100 | 107 | 111 | 2.1 | 2.3 | 3.7 | 4.2 |
| peak_clust_chr15_94.6mb | 9 | 1.0E+00 | 1.0E+00 | 1.0E+00 | 2.0E-02 | 1 | 3 | 2 | 7 | 119 | 72 | 100 | 116 | 0.5 | 3.3 | 1.3 | 13.1 |
| peak_clust_chr17_25.8mb | 88 | 7.9E-02 | 1.0E+00 | 1.0E+00 | 2.9E-01 | 60 | 15 | 31 | 50 | 228 | 95 | 157 | 186 | 3.0 | 0.8 | 1.1 | 2.0 |
| peak_clust_chr17_45.6mb | 81 | 2.5E-05 | 1.0E+00 | 1.9E-01 | 1.0E+00 | 56 | 14 | 40 | 45 | 182 | 124 | 150 | 242 | 4.5 | 0.6 | 2.1 | 1.2 |
| peak_clust_chr19_10.4mb | 20 | 9.5E-02 | 1.0E+00 | 1.0E+00 | 1.0E+00 | 10 | 7 | 0 | 3 | 98 | 98 | 46 | 92 | 4.6 | 2.4 | 0.0 | 0.9 |
| peak_clust_chr20_45.1mb | 38 | 1.0E+00 | 1.0E+00 | 1.0E+00 | 6.1E-06 | 4 | 7 | 2 | 31 | 36 | 125 | 40 | 188 | 1.7 | 0.7 | 0.7 | 7.9 |
| peak_clust_chr20_51.7mb | 37 | 1.0E+00 | 1.0E+00 | 1.0E+00 | 4.5E-04 | 3 | 8 | 4 | 29 | 35 | 128 | 42 | 203 | 1.3 | 0.9 | 1.4 | 5.7 |
| peak_clust_chr20_55.9mb | 35 | 1.0E+00 | 1.0E+00 | 1.0E+00 | 7.6E-03 | 3 | 11 | 4 | 28 | 30 | 130 | 65 | 233 | 1.6 | 1.4 | 0.9 | 5.0 |
| peak_clust_chr21_36.2mb | 8 | 4.2E-02 | 1.0E+00 | 1.0E+00 | 1.0E+00 | 5 | 2 | 1 | 3 | 73 | 103 | 70 | 117 | 10.9 | 1.5 | 1.0 | 2.2 |
