# Supplementary material for: Mutational mechanisms of amplifications revealed by analysis of clustered rearrangements in breast cancers
Source: Ann Oncol. 2018 Sep 25;29(11):2223–31. doi: 10.1093/annonc/mdy404 (PMC6290883; doi:10.1093/annonc/mdy404)
Supplement: Supplementary Data [file mdy404_supp.zip › mdy404-suppl_data/mdy404_Online_Supplementary_Notes.docx]

***Supplementary Notes***

***Supplementary Note 1: hotspots of rearrangements around oncogenes not typically associated with breast cancer***

Interestingly, several hotspots of clustered rearrangements were found near oncogenes that are not typically associated with breast cancer. Curation revealed that a subset had focal copy number gains typical of driver amplicons, albeit on a smaller scale (Supplementary Figure S4). These hotspots at or near *MCL1* (5.7% samples, 2.7% resulting in *MCL1* amplification), *PTP4A1* (4.5% samples, 1.25% *PTP4A1* amplification) and *MYB* (6.3%, 1.4% *MYB* amplification) occurred at lower frequencies than that of common breast cancer amplicons. Transcriptomic data was available for less than half of affected samples. Nevertheless, out of the three genes, clustered rearrangements in the hotspot close to the centromere of chromosome 6 were significantly associated with increased expression of PTP4A1 (P-value 0.03 in Wilcoxon test, Supplementary Figure S5). Although amplifications at the *MCL1*, *PTP4A1* and *MYB* loci are detectable in copy number profiles derived from array data (Nik-Zainal, 2016), their combined frequency and level of amplification across the breast cancer cohort had not reached the significance level of GISTIC analysis (Mermel et al., 2011). Thus, our method that is based on identifying recurrent clustered rearrangements provides supporting evidence that these more modestly amplified regions could be oncogenic drivers. We note that amplifications of these three loci had been observed in other cohorts, for example in the METABRIC cohort: MCL1 19%, PTP4A1 1.6%, MYB 3% ([Gao et al., 2013](#_ENREF_10); [Pereira et al., 2016](#_ENREF_14)). Further experiments will be required to verify whether these rarer, smaller and more modest amplicons are indeed driver events in the cancers that harbour them.

**References**

Nik-Zainal, S. (2016). A compendium of 560 breast cancer genomes. Nature.

Mermel, C. H. (2011). GISTIC2.0 facilitates sensitive and confident localization of the targets of focal somatic copy-number alteration in human cancers. Genome Biol.

Gao, J., Aksoy, B.A., Dogrusoz, U., Dresdner, G., Gross, B., Sumer, S.O., Sun, Y., Jacobsen, A., Sinha, R., Larsson, E.*, et al.* (2013). Integrative analysis of complex cancer genomics and clinical profiles using the cBioPortal. Sci Signal *6*, pl1.

Pereira, B., Chin, S.F., Rueda, O.M., Vollan, H.K., Provenzano, E., Bardwell, H.A., Pugh, M., Jones, L., Russell, R., Sammut, S.J.*, et al.* (2016). The somatic mutation profiles of 2,433 breast cancers refines their genomic and transcriptomic landscapes. Nat Commun *7*, 11479.
