# Supplementary material for: Mutational mechanisms of amplifications revealed by analysis of clustered rearrangements in breast cancers
Source: Ann Oncol. 2018 Sep 25;29(11):2223–31. doi: 10.1093/annonc/mdy404 (PMC6290883; doi:10.1093/annonc/mdy404)
Supplement: Supplementary Data [file mdy404_supp.zip › mdy404-suppl_data/mdy404_Supplementary_Table_1.docx]

| **Supplementary Table 1** |  |  |  |  |  |  |  |  |  |  |  |
| --- | --- | --- | --- | --- | --- | --- | --- | --- | --- | --- | --- |
| **hotspot.id** | ID of the hotspot | |  |  |  |  |  |  |  |  |  |
| **chr, start.bp, end.bp** | coodinates of hotspot | |  |  |  |  |  |  |  |  |  |
| **no.samples** | number of samples with clusters of rearrangements in the hotspots | | | |  |  |  |  |  |  |  |
| **noERPs, noTNs, noHER2s** | breakdown of samples according to cancer subtype | | |  |  |  |  |  |  |  |  |
| **bp.rate** | number of breakpoints in the hotspot, per basepair | | |  |  |  |  |  |  |  |  |
| **censusGenes** | cancer census genes in the hotspot | |  |  |  |  |  |  |  |  |  |
| **amplified.dom** | dominant cancer genes amplified (number of samples in bracket) | | | |  |  |  |  |  |  |  |
|  |  |  |  |  |  |  |  |  |  |  |  |
| **hotspot.id** | **chr** | **start.bp** | **end.bp** | **no.samples** | **noERPs** | **noTNs** | **noHER2s** | **bp.rate** |  | **censusGenes** | **amplified.dom** |
| peak_clust_chr11_65.1mb | 11 | 65113807 | 85296618 | 114 | 81 | 13 | 20 | 1.63E-04 |  | MAP3K11;CCND1;FADD;NUMA1;C11orf30;PAK1;GAB2 | PICALM(1)SF1(2)NUMA1(4)C11orf30(26)PAK1(30)GAB2(34)FADD(66)CCND1(69) |
| peak_clust_chr17_25.8mb | 17 | 25831516 | 39698236 | 88 | 28 | 7 | 53 | 1.61E-04 |  | NF1;SUZ12;TAF15;MLLT6;LASP1;CDK12;ERBB2;GRB7;IKZF3;CDC6;RARA;SMARCE1 | ETV4(1)STAT5B(3)STAT3(3)SUZ12(4)WSB1(7)TAF15(9)RARA(25)CDC6(27)MLLT6(27)LASP1(29)GRB7(53)ERBB2(54) |
| peak_clust_chr17_45.6mb | 17 | 45572444 | 80903837 | 81 | 29 | 7 | 45 | 1.49E-04 |  | NGFR;SPOP;COL1A1;HLF;MSI2;RNF43;CLTC;RPS6KB1;PPM1D;BRIP1;MAP3K3;CD79B;DDX5;AXIN2;BPTF;PRKAR1A;MAP2K6;SOX9;GRB2;H3F3B;SEPT9;CANT1;RNF213;RPTOR;ASPSCR1 | ETV4(1)ASPSCR1(5)CANT1(7)H3F3B(8)SEPT9(8)GRB2(11)MSI2(18)HLF(19)CD79B(20)DDX5(20)BPTF(26)COL1A1(30)NGFR(31)CLTC(34)RPS6KB1(35)PPM1D(36) |
| peak_clust_chr20_51.7mb | 20 | 51712677 | 55885546 | 37 | 26 | 0 | 11 | 1.39E-04 |  | ZNF217;AURKA | SS18L1(10)GNAS(12)ZNF217(27) |
| peak_clust_chr8_28.6mb | 8 | 28598705 | 43111249 | 76 | 49 | 11 | 16 | 1.14E-04 |  | WRN;BAG4;WHSC1L1;FGFR1;IKBKB;HOOK3 | HOOK3(21)MYST3(26)FGFR1(36)WHSC1L1(39) |
| peak_clust_chr21_36.2mb | 21 | 36225132 | 36789131 | 8 | 3 | 3 | 2 | 9.40E-05 |  | RUNX1 | ERG(1)RUNX1(2)OLIG2(2) |
| peak_clust_chr20_45.1mb | 20 | 45118773 | 51692499 | 38 | 27 | 1 | 10 | 8.79E-05 |  | NCOA3 | YWHAB(6)SDC4(6)NCOA3(12)ZNF217(26) |
| peak_clust_chr15_94.6mb | 15 | 94618067 | 100109555 | 9 | 8 | 0 | 1 | 8.21E-05 |  | IGF1R | CRTC3(1)IDH2(2)IGF1R(7) |
| peak_clust_chr20_55.9mb | 20 | 55896551 | 62909607 | 35 | 25 | 0 | 10 | 7.66E-05 |  | GNAS;SS18L1;BIRC7;EEF1A2;PTK6;ARFRP1 | BIRC7(9)EEF1A2(10)SS18L1(11)PTK6(11)GNAS(13)ZNF217(25) |
| peak_clust_chr1_201.3mb | 1 | 201280584 | 202202289 | 9 | 7 | 1 | 1 | 7.59E-05 |  |  | IKBKE(3)MDM4(5)SLC45A3(5)ELK4(6) |
| peak_clust_chr8_80.2mb | 8 | 80167433 | 130648936 | 80 | 32 | 19 | 29 | 6.51E-05 |  | HEY1;ZBTB10;ZNF704;NBN;RUNX1T1;MTDH;COX6C;YWHAZ;RRM2B;UBR5;RSPO2;RAD21;EXT1;NOV;MYC | RUNX1T1(20)MTDH(20)HEY1(20)COX6C(21)ZNF704(22)ZBTB10(24)NDRG1(25)YWHAZ(27)RRM2B(29)NOV(31)MYC(35) |
| peak_clust_chr12_65.1mb | 12 | 65118932 | 74024773 | 23 | 11 | 4 | 8 | 6.43E-05 |  | WIF1;HMGA2;MDM2;PTPRB | HMGA2(5)WIF1(5)MDM2(10) |
| peak_clust_chr6_63.3mb | 6 | 63279455 | 65643279 | 25 | 17 | 4 | 4 | 6.43E-05 |  | PTP4A1 | PTP4A1(7) |
| peak_clust_chr19_10.4mb | 19 | 10389906 | 11933465 | 20 | 12 | 2 | 6 | 6.22E-05 |  | KEAP1;DNM2;SMARCA4 | TPM4(0)MLLT1(1)NOTCH3(2)CALR(2)LYL1(3)BRD4(3) |
| peak_clust_chr1_60.8mb | 1 | 60823984 | 63240157 | 9 | 3 | 3 | 3 | 5.09E-05 |  |  | JAK1(1)JUN(1) |
| peak_clust_chr11_28.7mb | 11 | 28712359 | 38236914 | 25 | 12 | 8 | 5 | 4.25E-05 |  | WT1;LMO2 | LMO2(2)WT1(3) |
| peak_clust_chr6_117.6mb | 6 | 117646144 | 128392764 | 28 | 9 | 11 | 8 | 3.72E-05 |  | ROS1;GOPC;RSPO3;PTPRK | ROS1(2)GOPC(3) |
| peak_clust_chr6_96.6mb | 6 | 96552358 | 110767935 | 33 | 11 | 11 | 11 | 3.67E-05 |  | PRDM1;FOXO3 | FOXO3(3) |
| peak_clust_chr1_150mb | 1 | 150012451 | 165675659 | 32 | 17 | 9 | 6 | 3.65E-05 |  | MCL1;ARNT;MLLT11;TPM3;SHC1;CKS1B;MUC1;RAB25;PRCC;NTRK1;FCRL4;SDHC;FCGR2B;DDR2;PBX1 | NTRK1(3)FCRL4(4)PDE4DIP(4)PRRX1(5)RAB25(6)CHD1L(7)TPM3(7)PBX1(7)FCGR2B(9)BCL9(10)SHC1(11)CKS1B(11)MUC1(11)MLLT11(13)ARNT(13)MCL1(15) |
| peak_clust_chr12_0.1mb | 12 | 60710 | 2107516 | 10 | 4 | 4 | 2 | 3.62E-05 |  | KDM5A;ERC1 | CCND2(0)ZNF384(0)ERC1(1)KDM5A(1) |
| peak_clust_chr6_128.5mb | 6 | 128476393 | 153140319 | 35 | 15 | 10 | 10 | 3.53E-05 |  | PTPRK;MED23;MYB;MAP3K5;TNFAIP3;ECT2L;ESR1 | ESR1(2)MAP3K5(3)MYB(8) |
