# Supplementary material for: Mutational mechanisms of amplifications revealed by analysis of clustered rearrangements in breast cancers
Source: Ann Oncol. 2018 Sep 25;29(11):2223–31. doi: 10.1093/annonc/mdy404 (PMC6290883; doi:10.1093/annonc/mdy404)
Supplement: Supplementary Data [file mdy404_supp.zip › mdy404-suppl_data/mdy404_Supplementary_Table_2.docx]

| **Supplementary Table 2** | |  |  |  |  |  |  |
| --- | --- | --- | --- | --- | --- | --- | --- |
| **amp1, amp2** | combination of two amplifications | | |  |  |  |  |
| **amp1total** | number of samples with the first amplification | | | |  |  |  |
| **amp2total** | number of samples with the second amplification | | | |  |  |  |
| **noCoamp** | number of samples with both amplifications | | | |  |  |  |
| **oddsRatio** | odds ratio from the 2x2 table counting amplificaitons in two genes, separate and co-amplifications | | | | | | |
| **log10.pvalue** | P-value for number of observed coamplifications, assuming independece between amplifications of two genes | | | | | | |
| **p.value.norm** | P-value corrected for number of tests | | |  |  |  |  |
|  |  |  |  |  |  |  |  |
| **amp1** | **amp2** | **amp1total** | **amp2total** | **noCoamp** | **oddsRatio** | **log10.pvalue** | **p.value.norm** |
| ZNF703 | MYC | 64 | 110 | 20 | 1.86 | 1.69 | 2.05E-01 |
| ZNF703 | CCND1 | 64 | 88 | 24 | 3.22 | 6.03 | 1.86E-05 |
| ZNF703 | ERBB2 | 64 | 70 | 11 | 1.45 | 0.5 | 1.00E+00 |
| ZNF703 | ZNF217 | 64 | 36 | 11 | 3.02 | 3.26 | 8.70E-03 |
| MYC | ZNF703 | 110 | 64 | 20 | 1.72 | 1.69 | 2.05E-01 |
| MYC | CCND1 | 110 | 88 | 21 | 1.27 | 0.46 | 1.00E+00 |
| MYC | ERBB2 | 110 | 70 | 19 | 1.46 | 0.9 | 9.13E-01 |
| MYC | ZNF217 | 110 | 36 | 18 | 2.85 | 5.21 | 1.10E-04 |
| CCND1 | ZNF703 | 88 | 64 | 24 | 2.91 | 6.03 | 1.86E-05 |
| CCND1 | MYC | 88 | 110 | 21 | 1.28 | 0.46 | 1.00E+00 |
| CCND1 | ERBB2 | 88 | 70 | 16 | 1.56 | 0.94 | 9.13E-01 |
| CCND1 | ZNF217 | 88 | 36 | 12 | 2.3 | 2.25 | 7.94E-02 |
| ERBB2 | ZNF703 | 70 | 64 | 11 | 1.44 | 0.5 | 1.00E+00 |
| ERBB2 | MYC | 70 | 110 | 19 | 1.52 | 0.9 | 9.13E-01 |
| ERBB2 | CCND1 | 70 | 88 | 16 | 1.59 | 0.94 | 9.13E-01 |
| ERBB2 | ZNF217 | 70 | 36 | 10 | 2.43 | 2.04 | 1.10E-01 |
| ZNF217 | ZNF703 | 36 | 64 | 11 | 3.41 | 3.26 | 8.70E-03 |
| ZNF217 | MYC | 36 | 110 | 18 | 4.09 | 5.21 | 1.10E-04 |
| ZNF217 | CCND1 | 36 | 88 | 12 | 2.68 | 2.25 | 7.94E-02 |
| ZNF217 | ERBB2 | 36 | 70 | 10 | 2.69 | 2.04 | 1.10E-01 |
