# Supplementary material for: Mutational mechanisms of amplifications revealed by analysis of clustered rearrangements in breast cancers
Source: Ann Oncol. 2018 Sep 25;29(11):2223–31. doi: 10.1093/annonc/mdy404 (PMC6290883; doi:10.1093/annonc/mdy404)
Supplement: Supplementary Data [file mdy404_supp.zip › mdy404-suppl_data/mdy404_Online_Methods_Supplementary_information.docx]

***Methods – supplementary information***

***Dataset***

The primary dataset has been published earlier ([Nik-Zainal, 2016](#_ENREF_12)). Briefly, 560 matched tumour and normal DNAs were sequenced using Illumina sequencing technology, aligned to the reference genome and mutations called using a suite of somatic mutation calling algorithms as defined previously.

Somatic rearrangements were called using BRASS (https://github.com/cancerit/BRASS) for the discovery phase and then both in silico and /or PCR-based validation were performed in a subset of samples ([Nik-Zainal, 2016](#_ENREF_12)).

In the discovery phase, somatic rearrangements were called using discordantly mapping paired-end. Clipped reads were not used to inform discovery. Primary discovery somatic rearrangements were filtered against the germline copy number variants (CNV) in the matched normal, as well as a panel of fifty normal samples from unrelated samples to reduce the likelihood of calling germline CNVs and to reduce the likelihood of calling false positives.

The normal panel is one of the most effective filters at removing artefactual calls from:

- allele dropout of the matched normal,
- recurrent mapping errors that arise as a result of reference bias (e.g. polymorphic sites present in the population and not present in the reference),
- LINE-1 transductions.

In silico and /or PCR-based validation were performed in a subset of samples ([Nik-Zainal, 2016](#_ENREF_12)). Primers were custom-designed and potential rearrangements were PCR-amplified and identified as putatively somatic if a band observed on gel electrophoresis was seen in the tumour and not in the normal, in duplicate. Putative somatic rearrangements were then verified through capillary-sequencing. Amplicons that were successfully sequenced were aligned back to the reference genome using Blat, in order to identify breakpoints to basepair resolution. Alternatively, an *in silico* analysis was performed using local reassembly. Discordantly mapping read pairs that were likely to span breakpoints as well as a selection of nearby properly paired reads, were grouped for each region of interest. Using the Velvet de novo assembler, reads were locally assembled within each of these regions to produce a contiguous consensus sequence of each region. Rearrangements, represented by reads from the rearranged derivative as well as the corresponding non-rearranged allele were instantly recognisable from a particular pattern of five vertices in the de Bruijn graph (a mathematical method used in de novo assembly of (short) read sequences) of component of Velvet. Exact coordinates and features of junction sequence (e.g. microhomology or non-templated sequence) were derived from this, following aligning to the reference genome, as though they were split reads.

Only rearrangements that passed the validation stage, with rearrangement length over 1kb, were used in these analyses. Furthermore, additional post-hoc filters were included to remove library-related artefacts. A known library-preparation artefact was removed by filtering out inversions that were shorter than 5kb and reported by 5 or fewer reads.

RNA expression levels of genes in the samples were obtained from RNA-seq data together with PAM50 classifications, as reported previously ([Nik-Zainal, 2016](#_ENREF_12)).

# *The PCF algorithm*

The PCF (Piecewise-Constant-Fitting) algorithm is a method of segmentation of sequential data (Nilsson, 2008), (Nilsen, 2012) . Both breakpoints of each rearrangement were treated independently. The intermutation distance (IMD) between two genome-sorted rearrangement breakpoints was calculated for each breakpoint and log-transformed to base 10. Log 10 IMD were fed into the PCF algorithm. The parameters required for the PCF segmentation are: ***k_min_*** (minimum number of breakpoints in a segment) and ***γ*** (smoothness of segmentation).

Following the segmentation, a segment was declared a “hotspot” if the density of breakpoints contained exceeded the genome-wide density of breakpoints:

$$\frac{d_{seg}}{d_{bg}}>i$$

where $d_{seg}$is density of breakpoints in the segment and $d_{bg}$ is the genome-wide density of breakpoints. $i$ is the threshold determining how much the density of breakpoints in a segment exceeds the expected rate.

The choice of **k_min_**, ***γ*** and $\boldsymbol{i}$ was based on training against a simulated rearrangement dataset which corrected for rearrangement burden per sample, rearrangement type and rearrangement size. In the simulations, the position of the second breakpoint for each rearrangement was tied (by size) to its partner breakpoint.

We aimed to find as many hotspots as possible in the observed data while discovering as few in the simulated data as possible. This was quantified by calculating the false discovery rate. No hotspots of clustered rearrangements were found in the simulated data when the algorithm was set as follows: ***γ***= 16, **k_min_** =8 and $\boldsymbol{i=2}$**.**

***Workflow***

To define clustered from dispersed rearrangements in each sample, the PCF algorithm was applied to the IMD of each genome-sorted breakpoint per sample using the following parameters: ***γ*** = 25, **k_min_** = 8 and $\boldsymbol{i=10}$. If one of the breakpoints of a rearrangement falls into the cluster, the other breakpoint is also considered as clustered.

To define hotspots of clustered rearrangements recurrent across patients, the PCF algorithm was applied to the log10 IMD of pooled genome-sorted breakpoints of dispersed rearrangements using the following parameters: ***γ***= 16, **k_min_** =8 and $\boldsymbol{i=2}$. Post-hoc filtering was applied to reduce over-calling of hotspots and thus, only hotspots with a breakpoint from 8 samples or more were considered.

Throughout the analysis, a sample with at least one clustered rearrangement within the footprint of a hotspot would be counted as a sample with hotspot rearrangements. In the section on chromosome 6 however, to provide most reliable and conservative estimate of which hotspots are rearranged in particular samples, we modified the criterion. A hotspot in a sample is considered as rearranged if density of clustered breakpoints in the hotspot is significantly higher than chromosome-wide density of breakpoints, according to Binomial test.

***Copy number estimate from sequencing depth***

Copy number profiles (e.g. Figures 3 and 4) were estimated from local sequencing coverage in 10kb bins. The values were normalised with respect to GC content in the reference genome, as well as overall ploidy and cellularity as estimated by the ASCAT algorithm. Where whole chromosomes were shown, copy number bins were extended to 100kb.

***FISH design and staining for sample PD18733a***

The bacterial artificial chromosomal (BAC) probes were designed to lie as close to the estimated breakpoints as possible (Supplementary Figure S7). The BACs were selected from the UCSC Genome Browser and purchased as plasmid insert in E.Coli from Source Bioscience. The bacteria were cultivated according to the manufacturer instructions, and the plasmids were isolated with plasmid maxi kit (QIAGEN) and labelled with fluorescent dUTPs by nick translation (Nick translation kit, Invitrogen).

**References**

Nilsson, B., Johansson, M., Heyden, A., Nelander, S. & Fioretos, T. An improved method for detecting and delineating genomic regions with altered gene expression in cancer. Genome Biol 9, R13 (2008).

Nilsen, G. et al. Copynumber: Efficient algorithms for single- and multi-track copy number segmentation. BMC Genomics 13, 591 (2012).
