# Supplementary material for: Mutational mechanisms of amplifications revealed by analysis of clustered rearrangements in breast cancers
Source: Ann Oncol. 2018 Sep 25;29(11):2223–31. doi: 10.1093/annonc/mdy404 (PMC6290883; doi:10.1093/annonc/mdy404)

Supplementary Figure S1

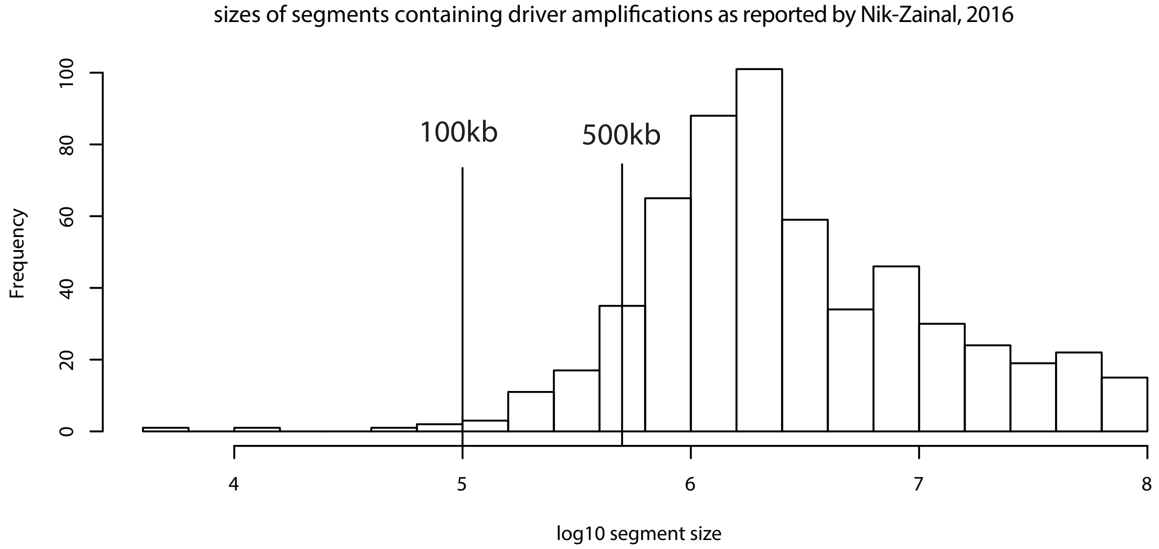

Supplementary Figure S2

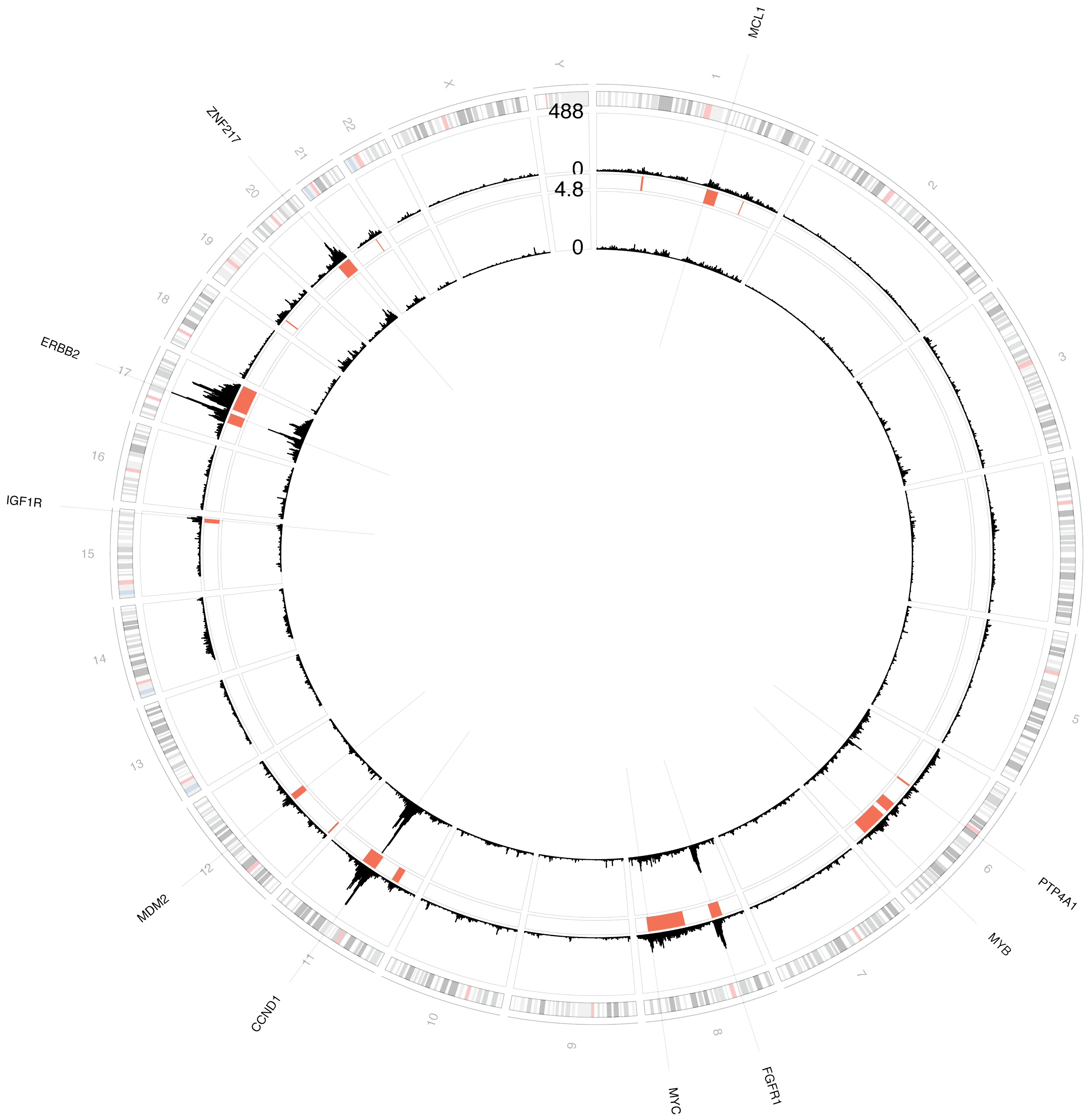

Supplementary Figure S3

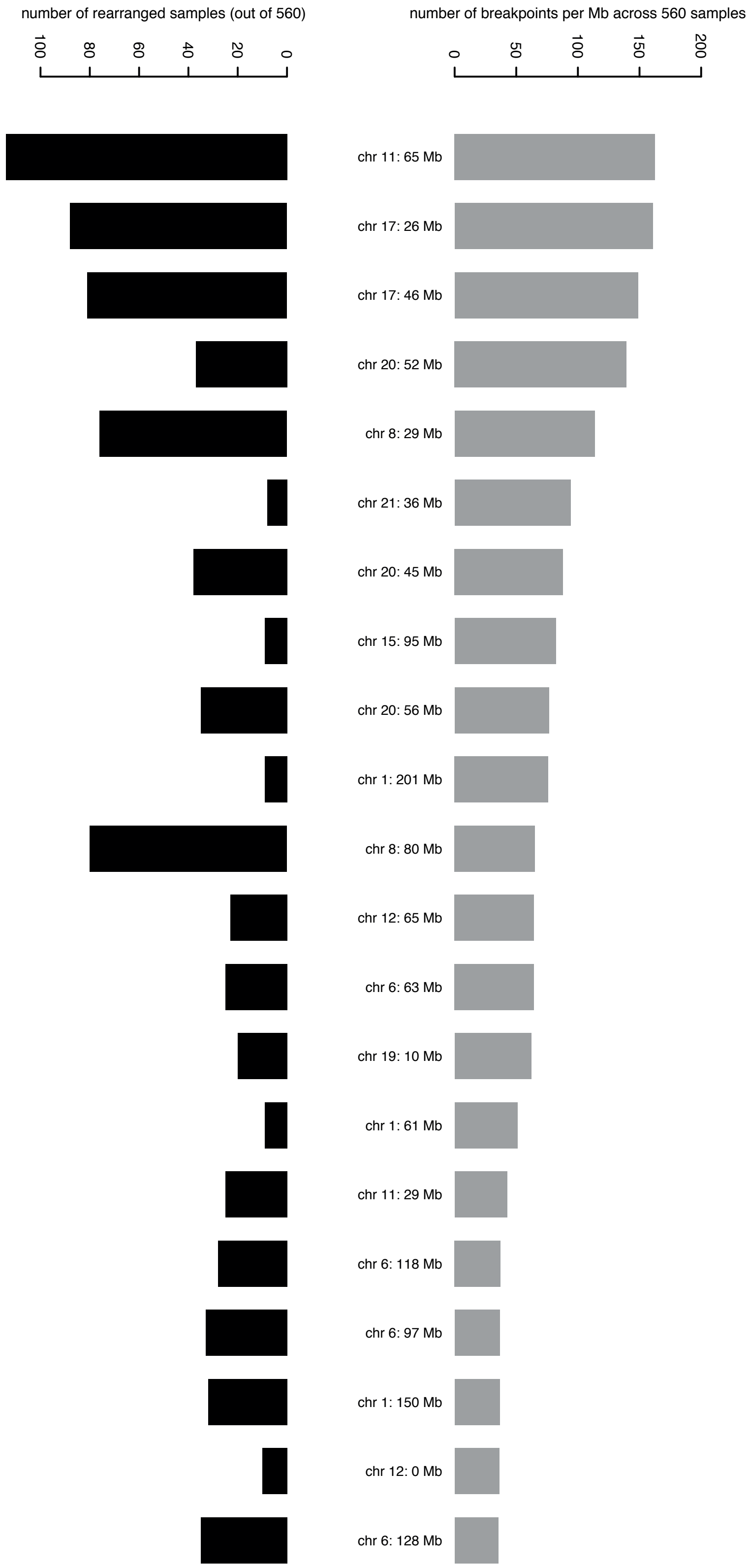

Supplementary Figure S4

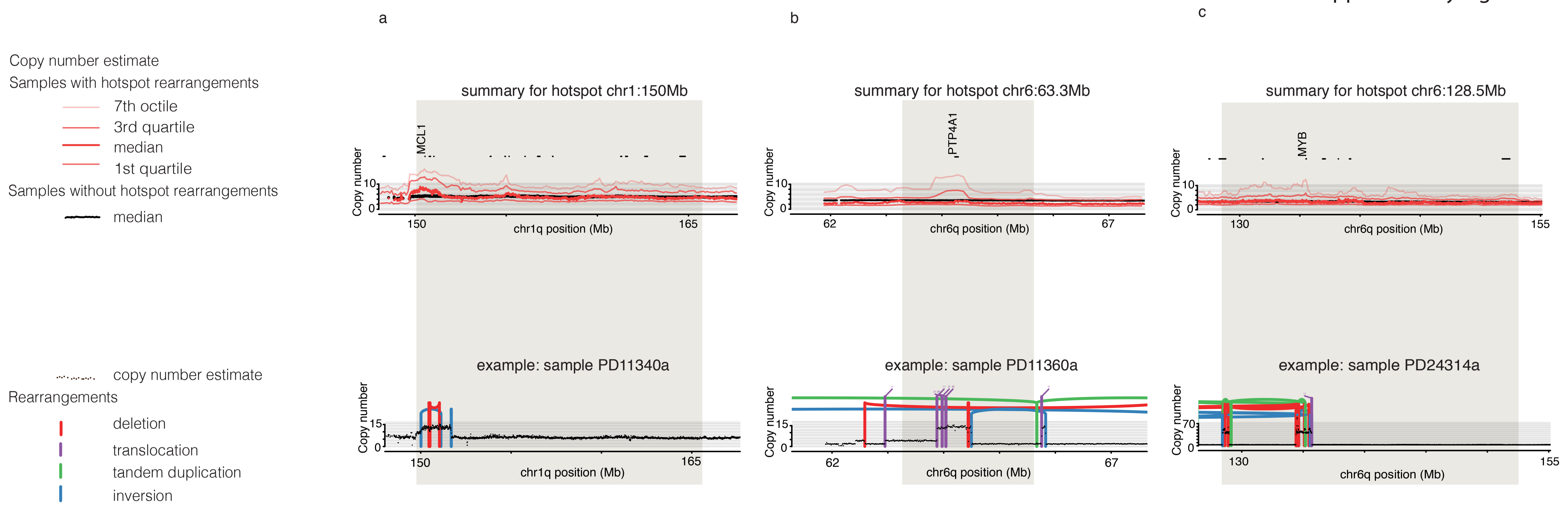

Supplementary Figure S5

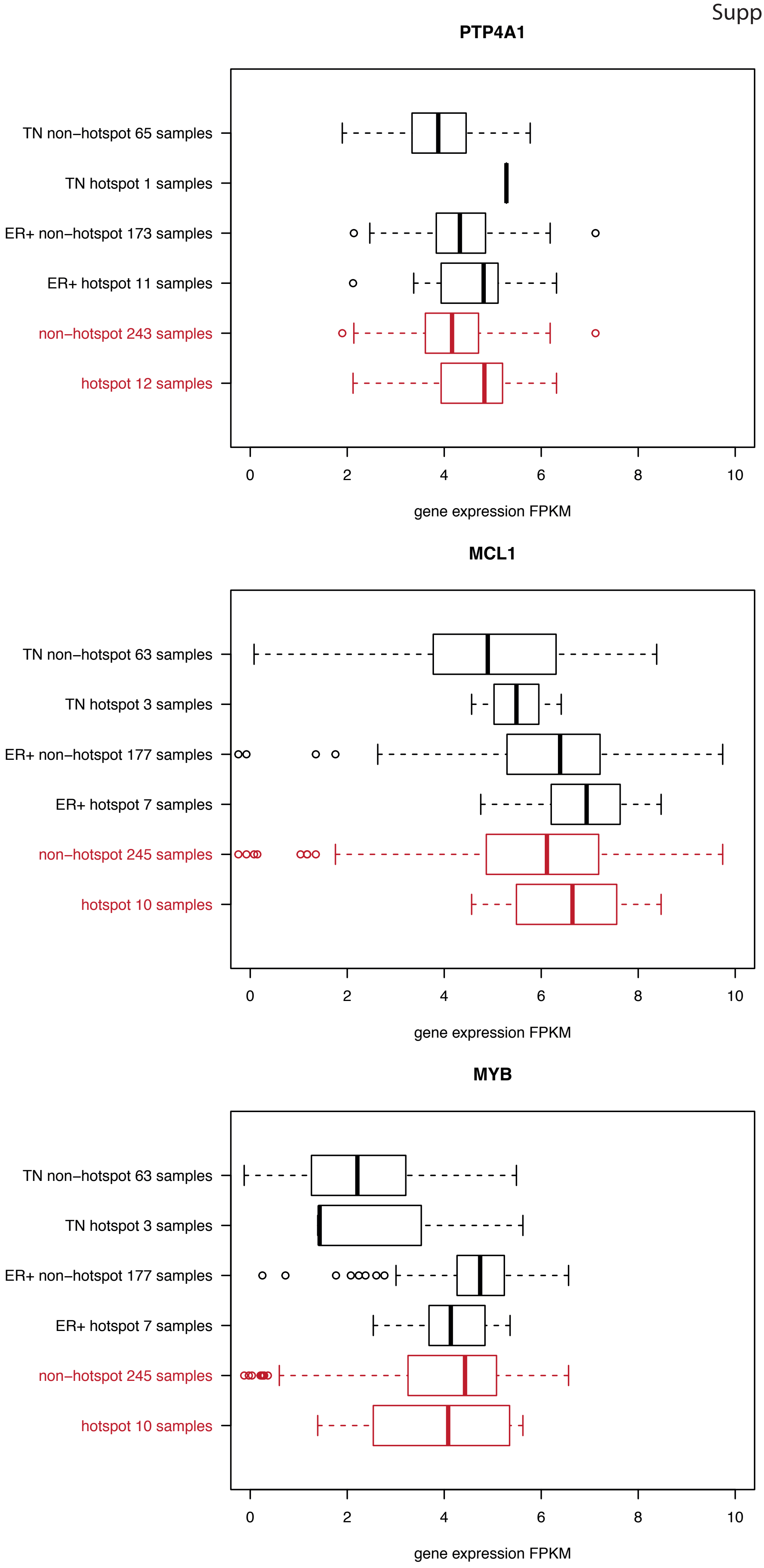

Supplementary Figure S6

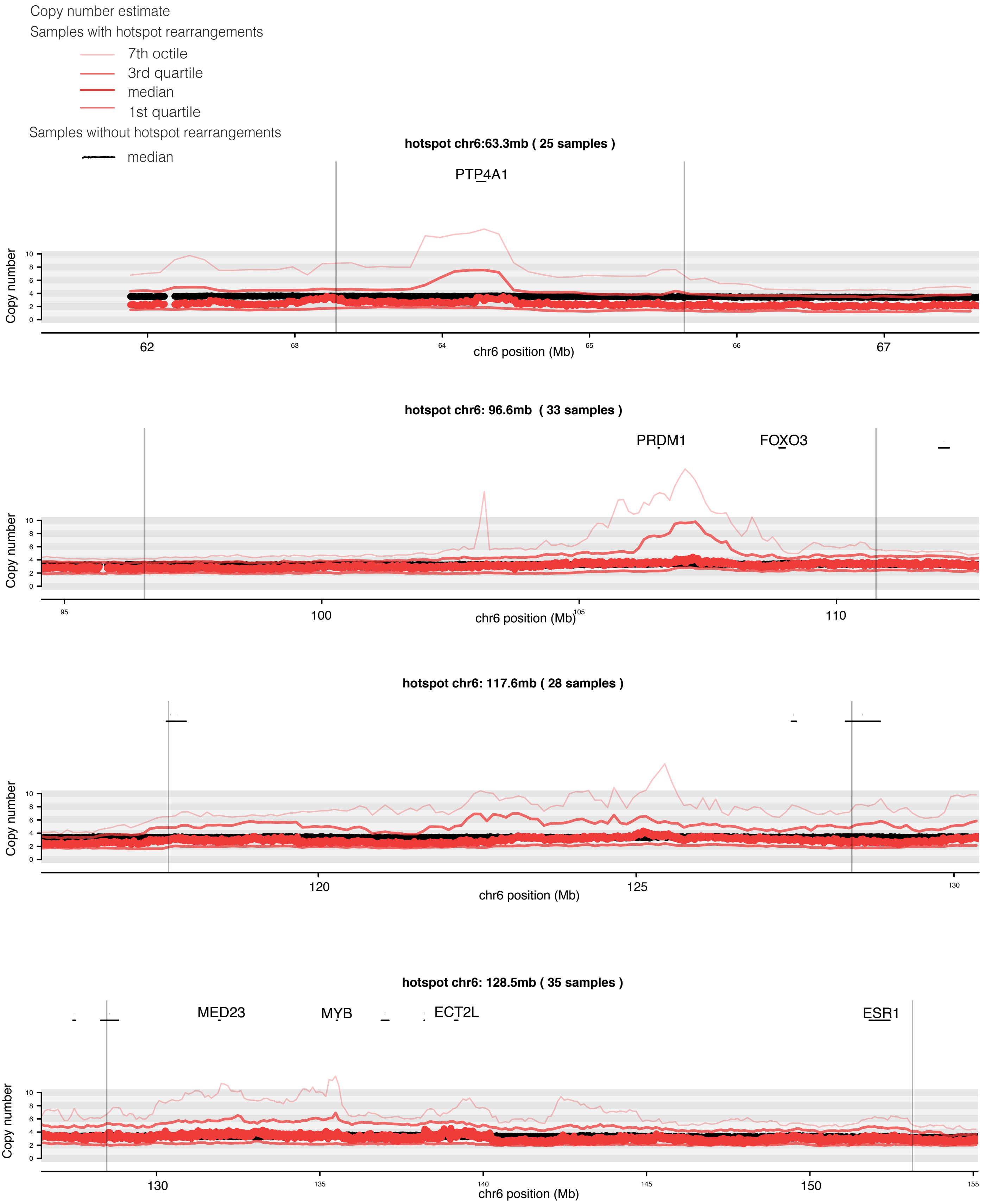

a

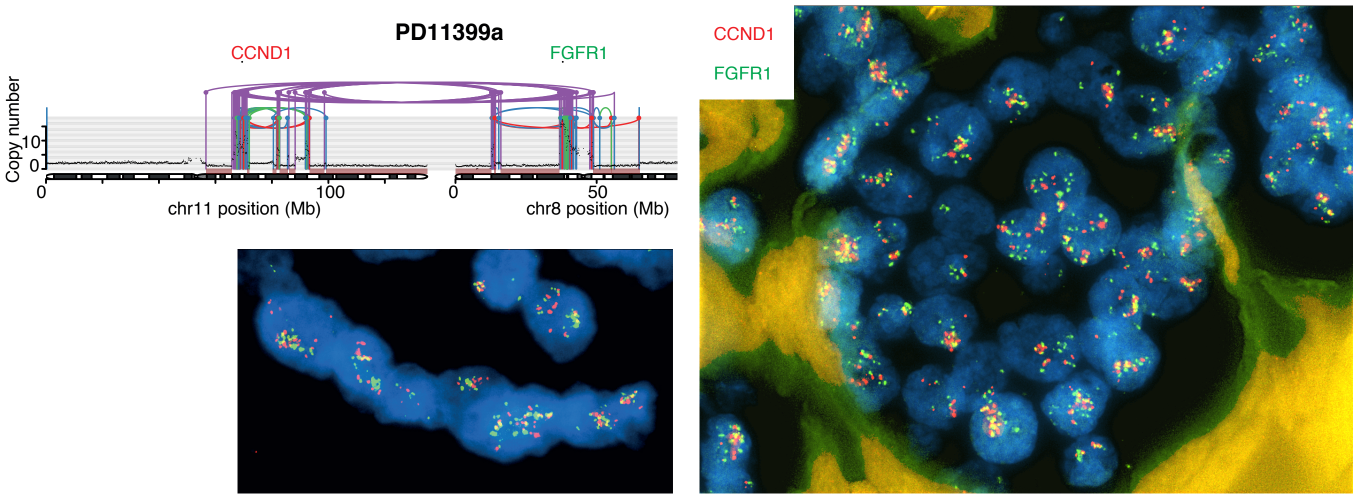

b

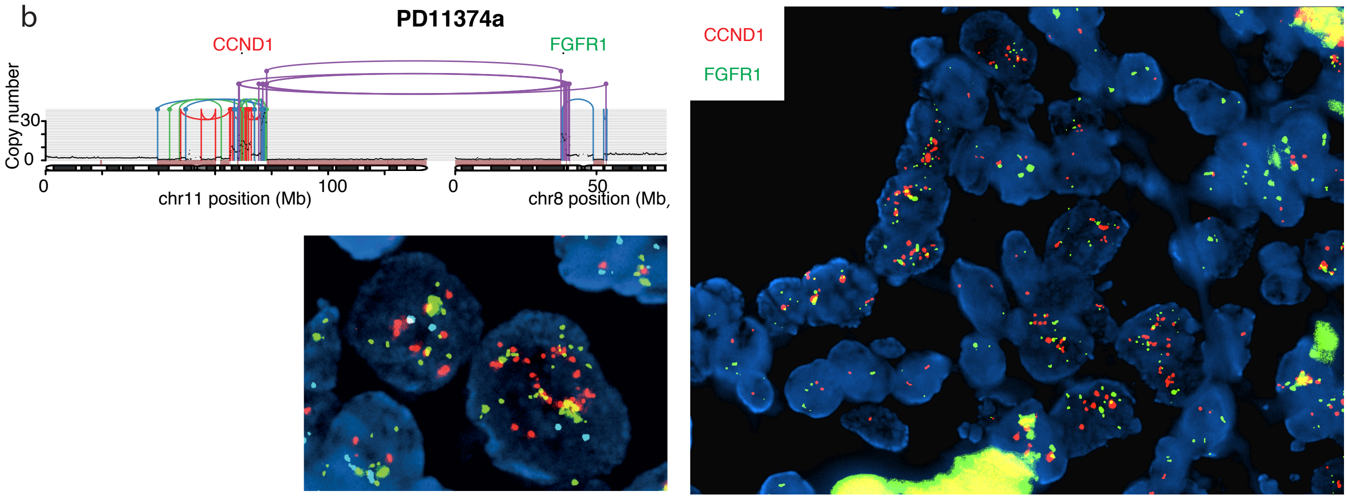

c

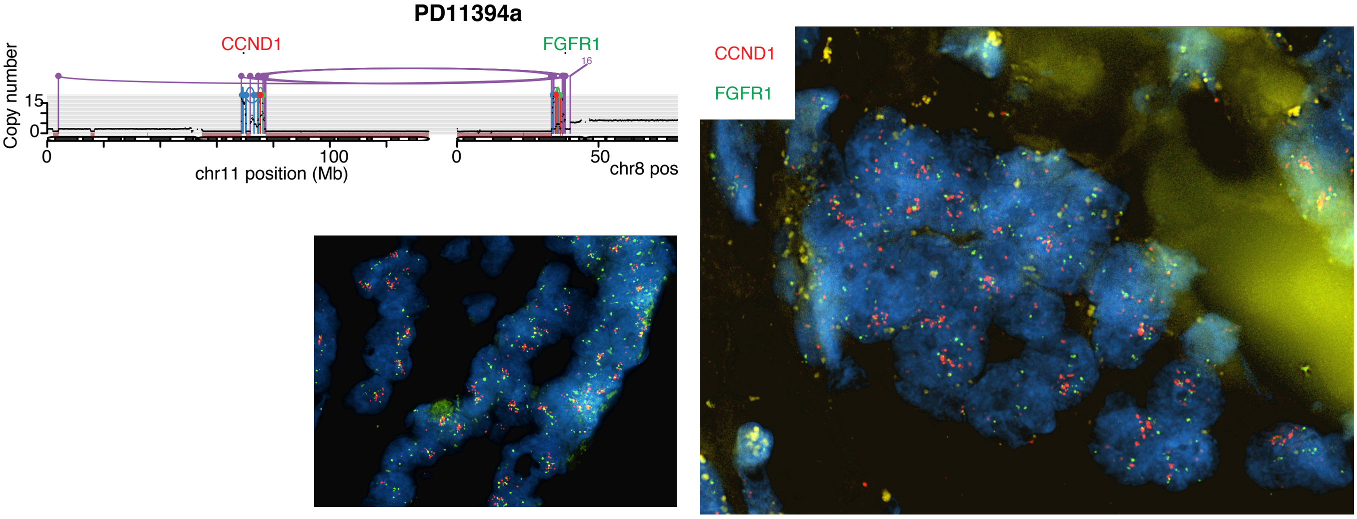

d

	Patient	Total FGFR1	Percentage of FGFR1 signals fused or close	Total CCND1	Percentage of CCND1 signals fused or close	Combined fused and close signals FGFR1-CCND1
PD11399a	DT8128b	365	290/365 = **80%**	353	290/353 = **82%**	290
	Average/cell	**7.0**	5.6	**6.8**	5.6	**5.6**
PD11374a	DT6758b	427	305/427 = **71%**	487	305/487 = **63%**	305
	Average/cell	**7.2**	5.2	**8.25**	5.2	**5.2**
PD11394a	DT7998b	150	115/150 = **77%**	265	115/265 = **43%**	115
	Average/cell	**5.0**	3.8	**8.8**	3.8	**3.8**

Supplementary Figure S8

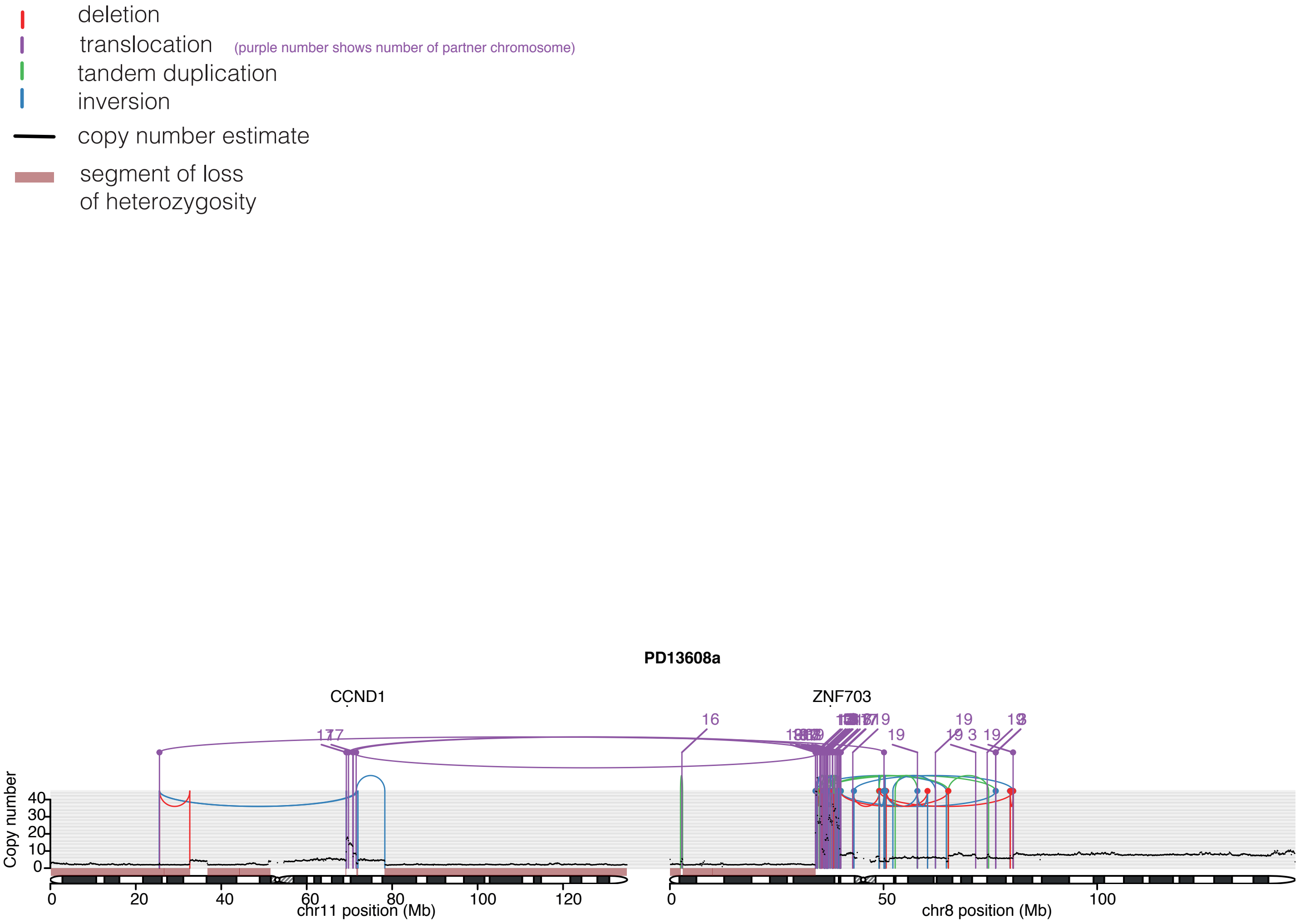

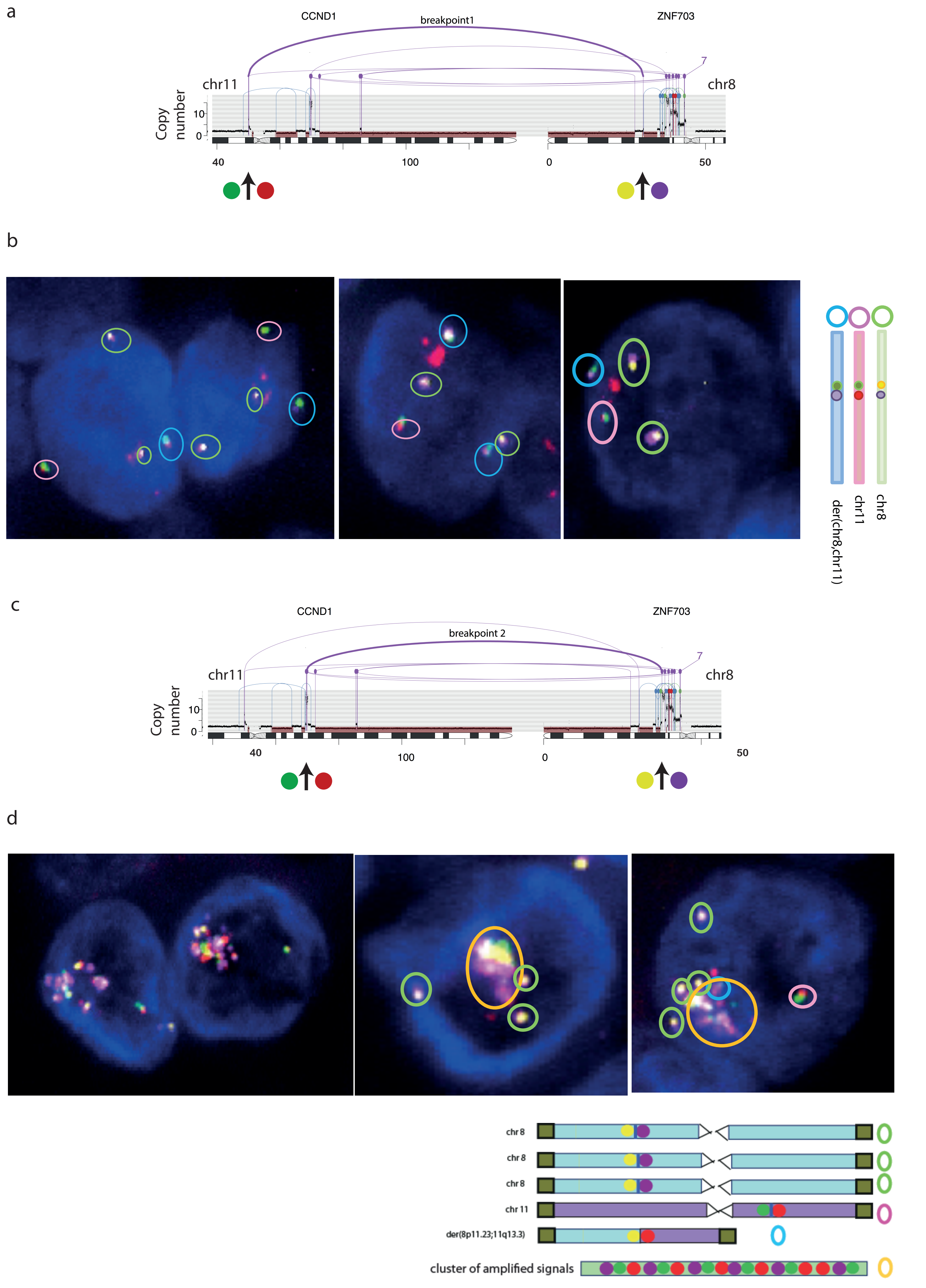
a
CCND1
breakpoint1
ZNF703
chr11
chr8
Copy number
10
0
40
100
0
50
b
der(chr8,chr11)
chr11
chr8
c
CCND1
breakpoint 2
ZNF703
chr11
chr8
Copy number
10
0
40
100
0
50
d
chr 8
chr 8
chr 8
chr 11
der(8p11.23;11q13.3)
cluster of amplified signals

Supplementary Figure S10

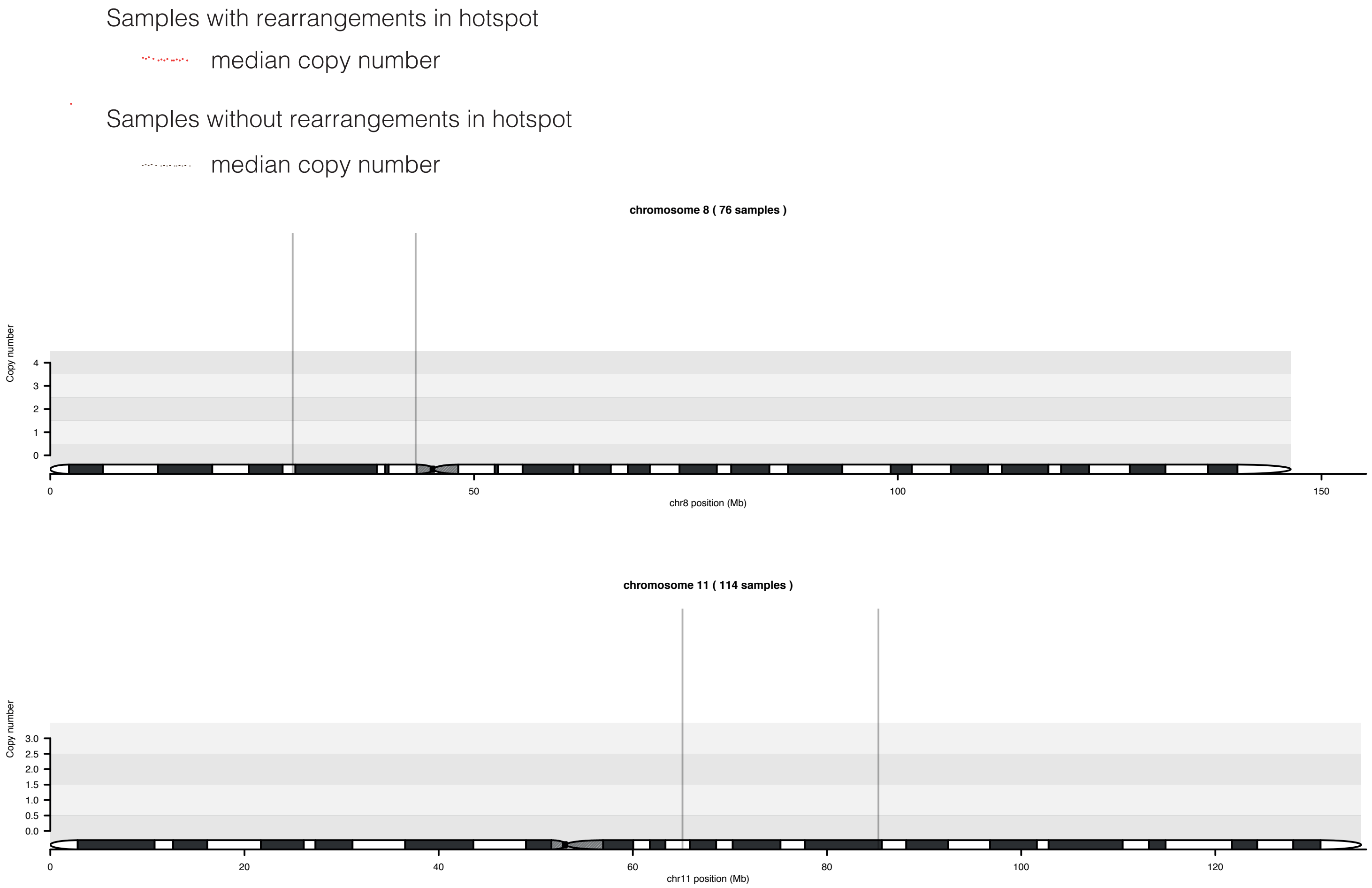

Supplementary Figure S11

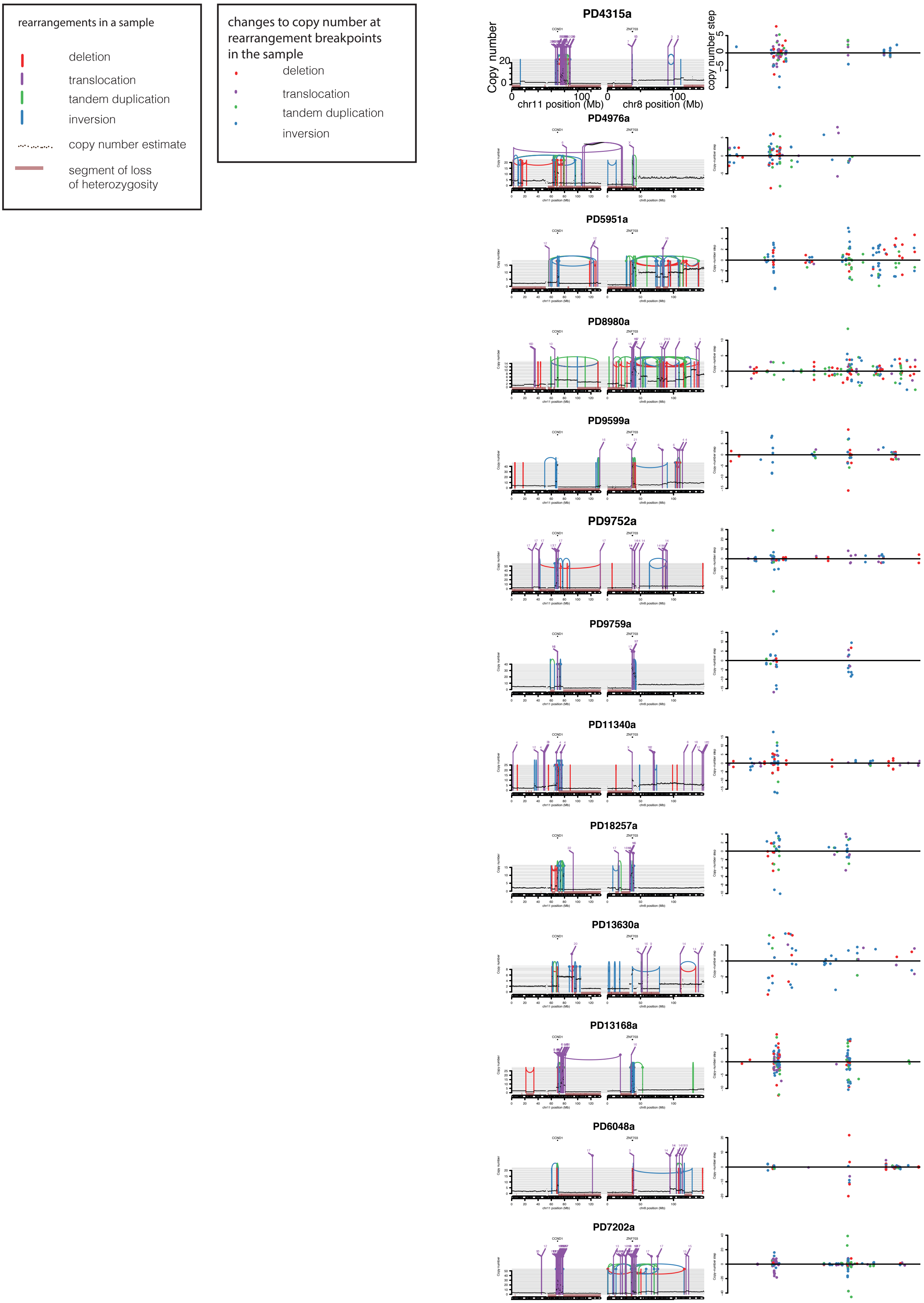

Supplementary Figure S12

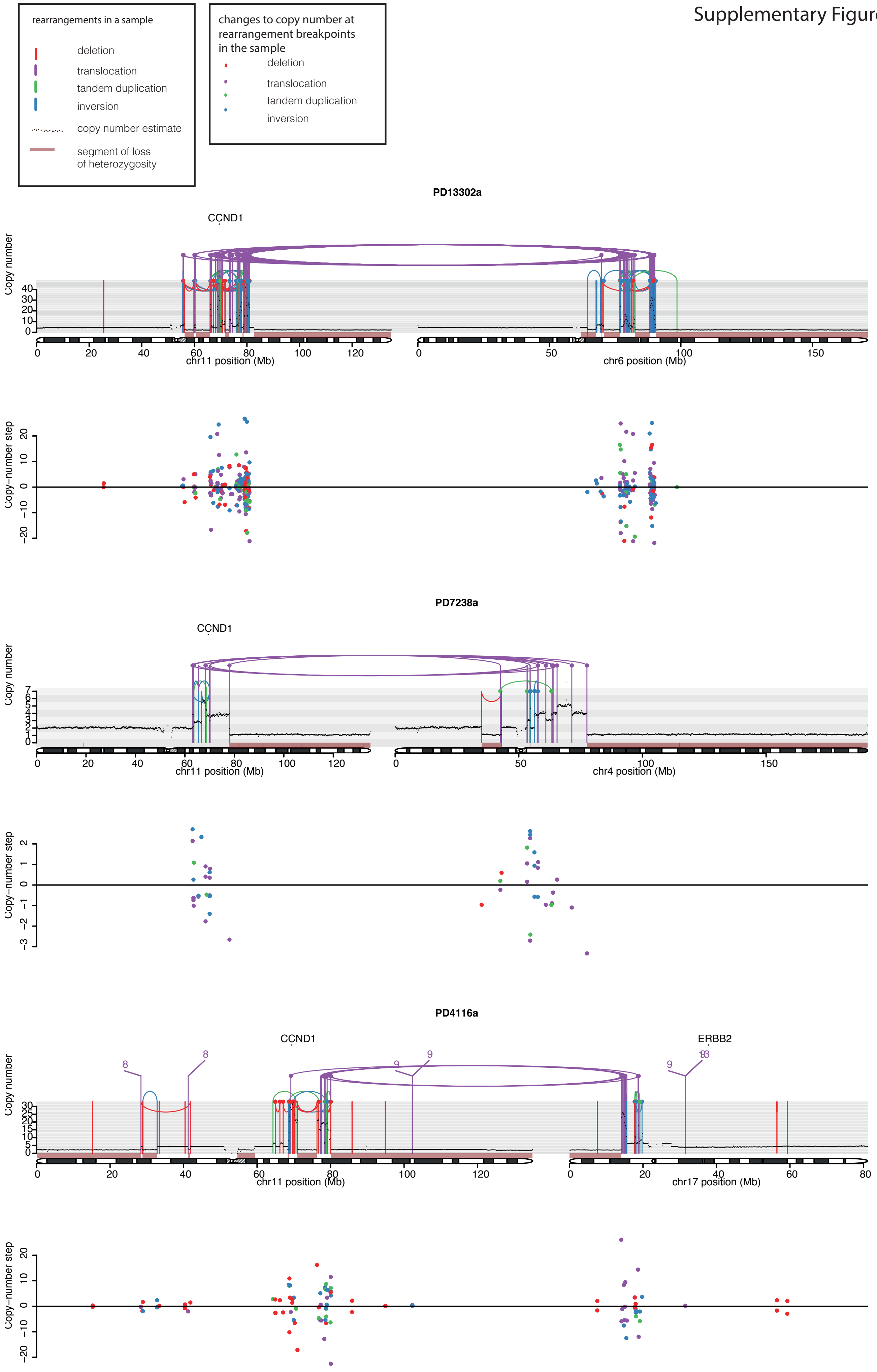

Supplementary Figure S13

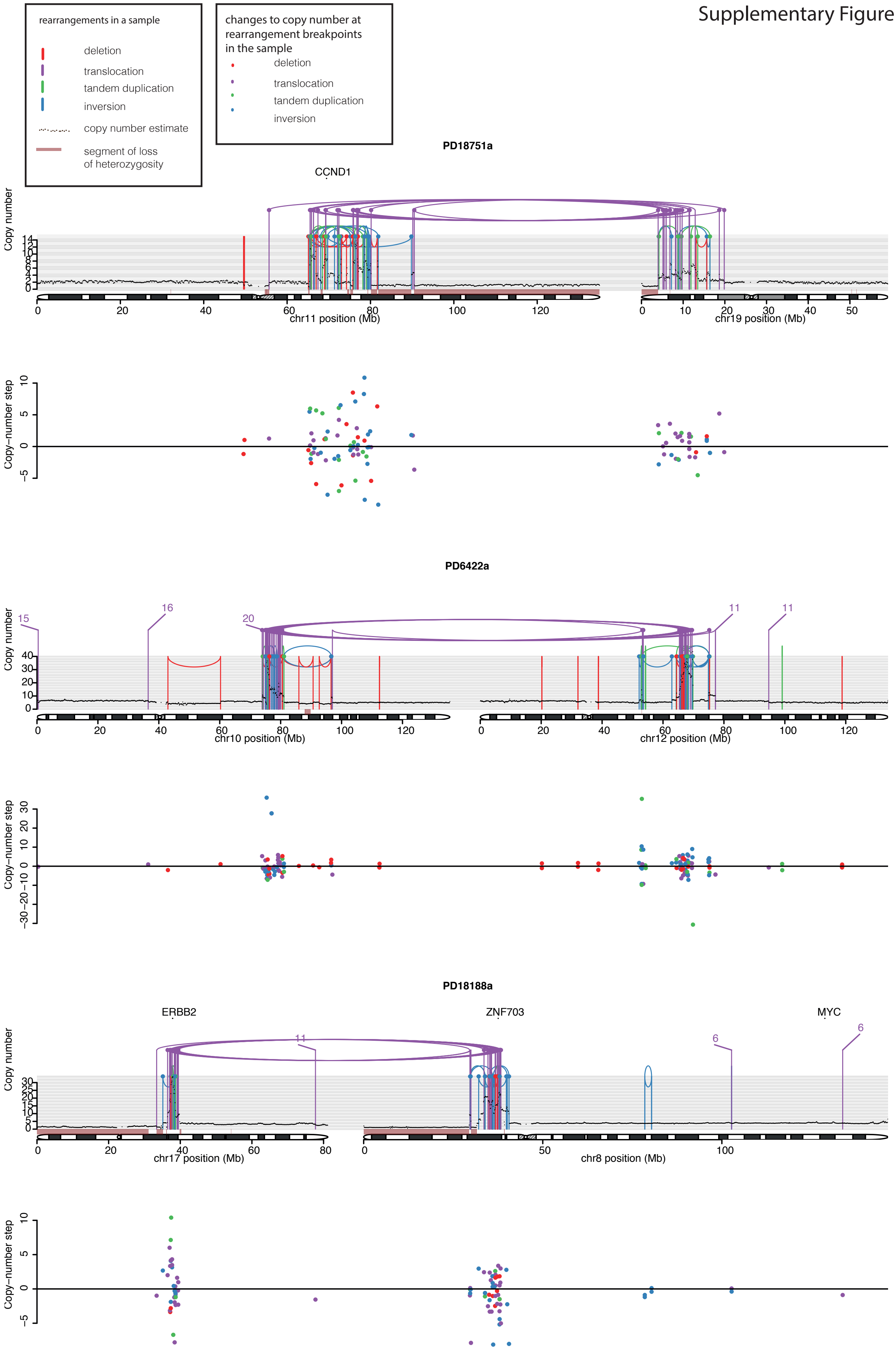

Supplement: Supplementary Data [file mdy404_supp.zip › mdy404-suppl_data/mdy404_All_Supplementary.pdf]
